# Atomic structure of the predominant GII.4 human norovirus capsid reveals novel stability and plasticity

**DOI:** 10.1038/s41467-022-28757-z

**Published:** 2022-03-10

**Authors:** Liya Hu, Wilhelm Salmen, Rong Chen, Yi Zhou, Frederick Neill, James E. Crowe, Robert L. Atmar, Mary K. Estes, B. V. Venkataram Prasad

**Affiliations:** 1 Verna and Marrs McLean Department of Biochemistry and Molecular Biology, Baylor College of Medicine, Houston, TX USA; 2 Department of Molecular Virology and Microbiology, Baylor College of Medicine, Houston, TX USA; 3 The Vanderbilt Vaccine Center, Vanderbilt University Medical Center, Nashville, TN USA; 4 Department of Pediatrics, Vanderbilt University Medical Center, Nashville, TN 37232 USA; 5 Section of Infectious Diseases, Department of Medicine, Baylor College of Medicine, Houston, TX USA; 6 Section of Gastroenterology and Hepatology, Department of Medicine, Baylor College of Medicine, Houston, TX 77030 USA

**Keywords:** X-ray crystallography, Cryoelectron microscopy

## Abstract

Human noroviruses (HuNoVs) cause sporadic and epidemic viral gastroenteritis worldwide. The GII.4 variants are responsible for most HuNoV infections, and GII.4 virus-like particles (VLPs) are being used in vaccine development. The atomic structure of the GII.4 capsid in the native *T* = 3 state has not been determined. Here we present the GII.4 VLP structure with *T* = 3 symmetry determined using X-ray crystallography and cryo-EM at 3.0 Å and 3.8 Å resolution, respectively, which reveals unanticipated novel features. A novel aspect in the crystal structure determined without imposing icosahedral symmetry is the remarkable adaptability of the capsid protein VP1 driven by the flexible hinge between the shell and the protruding domains. In both crystal and cryo-EM structures, VP1 adopts a stable conformation with the protruding domain resting on the shell domain, in contrast to the ‘rising’ conformation observed in recent cryo-EM structures of other GII.4 VLPs. Our studies further revealed that the resting state of VP1 dimer is stabilized by a divalent ion, and chelation using EDTA increases capsid diameter, exposing new hydrophobic and antigenic sites and suggesting a transition to the rising conformation. These novel insights into GII.4 capsid structure, stability, and antigen presentation may be useful for ongoing vaccine development.

## Abstract (web summary)

GII.4 variants of human noroviruses cause pandemic viral gastroenteritis. The atomic structure of GII.4 virus-like particles reveal novel insights into capsid stability, dynamics and antigenic presentation potentially useful for ongoing vaccine development.

## Introduction

Noroviruses (NoVs), belonging to the genus *Norovirus* in the *Caliciviridae* family, are nonenveloped, positive-sense single-stranded RNA viruses capable of infecting many mammalian hosts, including humans, dogs, cats, pigs, mice, sheep, and cattle^1^. In humans, these viruses (HuNoVs) are the causative agents of epidemic and sporadic acute viral gastroenteritis worldwide, resulting in ~684 million illnesses and ~212,000 deaths annually^2–4^. The genome of NoVs is ~7.5 kb in length and consists of three open reading frames (ORFs). ORF1 codes for six non-structural proteins that are absent in the mature viral particles but play critical roles during virus replication. ORF2 encodes the major capsid protein VP1 that encapsidates the (+) sense ssRNA genome and ORF3 codes for the minor structural protein VP2 that is suggested to be located internally in the capsid^5^.

Based on the amino acid sequences of VP1, NoVs are phylogenetically classified into at least ten genogroups (GI-GX), which are further subdivided into 49 genotypes (e.g., GII.4)^6^. NoVs of GI, GII, GIV, GVIII, and GIX genogroups infect humans, and the genotype GII.4 has been the predominant NoV genotype accounting for up to 80% of HuNoV infections. HuNoVs are highly contagious, and a few viral particles can lead to infection. Young children, immunocompromised patients, and the elderly are especially at high risk. Although there are candidate vaccines based on HuNoV virus-like particles (VLPs) in clinical development^7,8^, as yet there are no approved vaccines or antivirals for prevention or treatment. It remains unknown whether the VLP-based vaccines can provide long-term immunity against both homotypic and heterotypic infection with various epochally evolving GII.4 HuNoVs that are genetically and antigenically distinct^9^. A deeper understanding of the capsid structures may provide a rational basis for developing more effective vaccines.

**Fig. 1:**
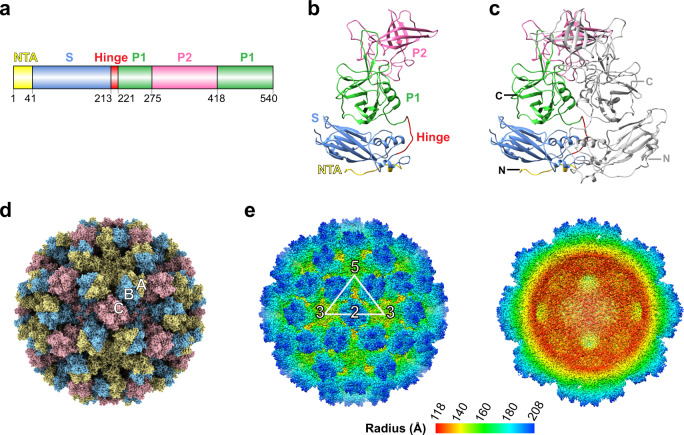
Crystal structure of GII.4 HOV VLP. **a** Schematic of VP1 primary structure colored by domain. **b** Ribbon representation of VP1 monomer. The NTA, S domain, hinge, P1 subdomain, and P2 subdomain are colored corresponding to the schematic (**a**). **c** The VP1 dimeric formation. One subunit is colored as in **b**, and the other subunit is shown in gray. The N- and C- termini of the structure are labeled. **d** GII.4 HOV VLP structure viewed along the icosahedral twofold axis. The subunit A, B, and C are colored yellow, blue, and pink. **e** The surface and cut-away views of a GII.4 crystal surface colored by radial distance showing an inner diameter of ~ 230 Å and an outer diameter of ~ 410 Å.

Despite the recent success in the in vitro cultivation of HuNoVs using stem cell-derived human enteroids^10,11^, it is still challenging to obtain enough native viral particles for structural studies. Therefore, VLPs, which are antigenically and structurally similar to native virions but lack the genome and are formed by recombinant expression of the capsid protein VP1 with or without the minor structural protein VP2, have been extensively used as surrogates to characterize the structure of HuNoV viral particles^12–14^. The first structure of a GI.1 VLP capsid was determined using X-ray crystallography^13^. Following this, X-ray crystallographic structures of the native calicivirus of animal origins such as feline calicivirus and San Miguel sea lion virus, and cryo-EM structures of rabbit hemorrhagic disease virus and receptor-bound feline calicivirus have been determined to near-atomic resolution^15–18^. All these structures showed that the capsid exhibits T = 3 icosahedral symmetry, with a diameter of ~400 Å, composed of 90 dimers of the capsid protein VP1 that consists of an internal N-terminal arm (NTA) and two distinct domains separated by a flexible hinge (Fig. 1a–c). In the capsid architecture, the N-terminal domain ‘ties’ the VP1 subunits through a network of interactions facing the interior of the capsid, the S domain forms the icosahedral shell, and the P domain protrudes from the S domain. The P domain is further divided into P1 and P2 subdomains. The distally located P2 subdomain, which shows significant sequence variation among HuNoVs, is implicated in strain-dependent specific recognition of cellular glycans that function as both cell attachment and susceptibility factors^19^.

More recently, cryo-EM structures of various HuNoV VLPs representing GI.1, GI.7, GII.2, GII.4 HuNoV VLPs have been determined^12,14,20^. These structures reveal significant variations in the molecular organization of the capsid (Supplementary Table 1). While GI.1 and GI.7 VLPs exhibit T = 3 icosahedral symmetry consisting of 180 copies of VP1, the GII.2 VLP assembles into T = 1 and T = 3 symmetries. Some GII VLPs, including GII.4 variants (Minerva, CHDC-1974, NSW-2012 and the vaccine candidate GII.4c), show T = 4 symmetric organization, with a diameter of ~500 Å, composed of 240 VP1 subunits. In addition, these studies showed that VP1 could exist in two distinct conformations similar to what was observed for VP1 of murine NoV^21–23^. In one conformation, the P domain rests on the S domain, referred to as a ‘resting’ conformation, whereas in the other orientation, it is rotated and elevated from the S domain, called a ‘raised’ conformation.

The infectious GII.4 virion, similar to various animal calicivirus, has a smaller diameter of ~400 Å that is consistent with a T = 3 symmetry using negative stain EM^20,24^. Although the cryo-EM structure of a GII.4 variant Minerva VLP with a T = 4 symmetry at 4.1 Å has been determined, the atomic structure of a GII.4 HuNoV VLP capsid with T = 3 symmetry as the native virus is not reported. Here we report the atomic structure of a GII.4 VLP determined using X-ray crystallography and cryo-EM at 3.0 Å and 3.8 Å resolution, respectively. In both structures, the GII.4 capsid exhibits the native T = 3 symmetry. A unique aspect of our studies is that the crystallographic structure of the VLP is determined without explicitly imposing icosahedral symmetry, which is rather unprecedented and allowed us to probe into conformational flexibility of the capsid protein that is permissible within the context of an icosahedrally symmetric organization. In addition to a detailed description of how GII.4 VP1 adapts to various quasiequivalent positions on a T = 3 icosahedral lattice both in the crystalline state and in solution, our studies revealed novel features, including identification of an ion-binding site that could impinge upon the stability and conformational flexibility of the GII.4 T = 3 capsid that may play a role in the cell entry processes and therefore provide informed strategies for vaccine development.

## Results

### Crystal structure of GII.4 VLP shows a native T = 3 capsid organization

To elucidate the molecular details of the GII.4 VLP (HOV, 2002 strain) structure, we expressed the capsid proteins VP1 and VP2 using the baculovirus expression system. Previous studies have shown that the co-expression of VP1 and VP2 results in stable and structurally homogenous particles^25^. We initially determined the crystal structure of GII.4 HOV by imposing icosahedral symmetry using the protocols previously described^13,15,16^. Although the electron density representing the shell was clearly depicted, the electron density for the distal parts of the capsid was poor, indicating that parts of the capsid structure deviated from strict icosahedral symmetry, perhaps affected by the packing of the GII.4 VLP in the crystal with the I222 space group. We then resorted to determining the structure using only the non-crystallographic symmetry present within the crystallographic asymmetric unit (Supplementary Table 2). The crystallographic asymmetric unit in the I222 space group contains 45 copies of the capsid protein VP1. The molecular replacement using GI.1 Norwalk VLP as the search model revealed the locations of all the 45 VP1 subunits in the asymmetric unit (Supplementary Fig. 1a). These subunits were refined using non-crystallographic symmetry restraints without imposing the icosahedral 532 symmetry. The four asymmetric units of the unit cell related by the crystallographic 222 symmetry forms the complete capsid structure consisting of 180 VP1 subunits with all the characteristics expected in a T = 3 organization (Supplementary Fig. 1b). For further analysis, these subunits were classified as 60 sets of traditionally designated A, B, and C quasi-equivalent subunits representing the icosahedral asymmetric unit in a T = 3 lattice (Fig. 1d, e). These subunits cluster into 60 AB and 30 CC dimers in the capsid. The GII.4 VLP has an outer diameter of ∼410 Å and an inner diameter of ∼230 Å (Fig. 1e). In the final model, following iterative cycles of refinement and model building, residues 26–531 of the A and C subunit and residues 10–532 of the B subunit were visualized. Although the minor structural protein VP2 was co-expressed with VP1 to obtain the VLPs, no electron density accounting for VP2 residues was observed. This might be because of low copy numbers (1.5 - 8 copies) of VP2 per viral particle^26,27^, the intrinsic structural flexibility/disorder in VP2, or the lack of specific strong interactions with the VP1 subunits.

**Fig. 2:**
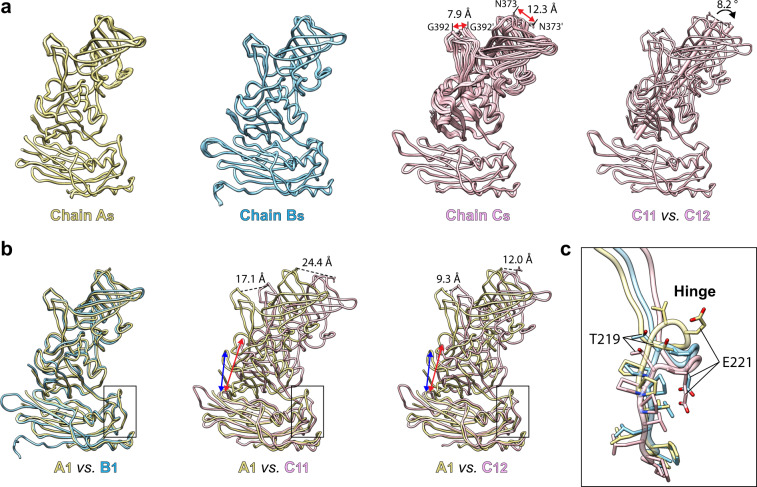
Structural comparison of VP1 subunits revealing its intrinsic flexibility. **a** Superimposition of 15 copies of subunit A, B, or C, respectively. The structural change between Cs is indicated by red double-arrows. The distances between Cα atoms of residue G392 and the side chains of N373 are measured and shown, respectively. **b** Superimposition of subunit A with B or C subunits using the S domain. The distance between a β-stand of P and S domains is indicated by blue (subunit A) and red (subunit C) double-arrows. The distances between Cα atoms of residue G392 and the side chains of N373 are measured and shown, respectively, as in Fig. 2a. The black box indicates the hinge region of VP1. **c** Close-up view of the hinges of A, B, and C chains. The stick model of residues 213–221 is shown and colored as in a.

As expected, the structure of each VP1 subunit comprises the NTA, the S domain, and the P domain (Fig.1b, c). The S domain (residue 41–213) has an eight-stranded β-barrel jellyroll structure and forms the icosahedral shell. The P domain, linked to the S domain by a flexible hinge, comprises residues 222–540. The P domain is further divided into P1 and P2 subdomains. The P1 subdomain, consisting of two segments of the polypeptide (residues 222–275 and 419–540), folds into a structure similar to GI.1 Norwalk virus and other GII.4 P domain crystal structures. The S domains of the AB and CC dimers show bent and flat conformations to impart the required curvature to form the icosahedral shell. Although there are small differences, as detailed separately below, in all the subunits the P domain closely interacts with the S domain (Fig. 2) (‘resting’ conformation) in stark contrast to what is observed in the T = 4 icosahedral structure of a GII.4 variant in which the P domain lacks any of the interactions with the S domain and is raised above by ~20 Å.

### The intrinsic flexibility of VP1 subunits

The X-ray structure of the GII.4 VLP without explicitly imposing the icosahedral symmetry allowed us to probe into the intrinsic flexibility in the capsid protein within the crystallographic asymmetric unit containing 15 copies of the A, B, and C subunits. We wanted to address whether these 15 copies maintained identical conformations, as would be expected in the T = 3 capsid with strict icosahedral symmetry, or whether they showed structural alterations, thereby providing insight into the allowed flexibility within the T = 3 organization. Although the overall conformation of the S and P domains in these subunits is maintained, pairwise superposition of A, B, and C subunits in the crystallographic asymmetric unit showed that while the subunits A and B superimposed well with RMSD of 0.4 Å and 0.3 Å, respectively, the C subunits showed significant variations with an RMSD of 1.2 Å (Fig. 2a). In all the A and B subunits, the P domain making close contact with the S domain is oriented in a similar manner; however, in the C subunits, as a result of the conformational changes in the flexible hinge, there are noticeable alterations in the orientation between the S and P domains (Fig. 2b, c). Examination of the crystal packing shows several of the C subunits in the capsid make close contact with the neighboring capsid in the crystal and the flexible hinge allows their P domains to suitably accommodate such a crystal packing (Supplementary Fig. 1c). Interestingly, the icosahedral symmetry at the level of the S domain is well maintained as well as the 5-fold symmetry that relates the AB dimers. These observations indicate that to maintain the integrity of the T = 3 architecture, proper interactions between the S domains are critical and that the A-B dimers surrounding the 5-fold axes have to be conformationally more restrained; however, the C-C dimers can deviate from the strict icosahedral symmetry.

### Cryo-EM structure shows GII.4 VLP adopts the same T = 3 capsid assembly in solution

**Fig. 3:**
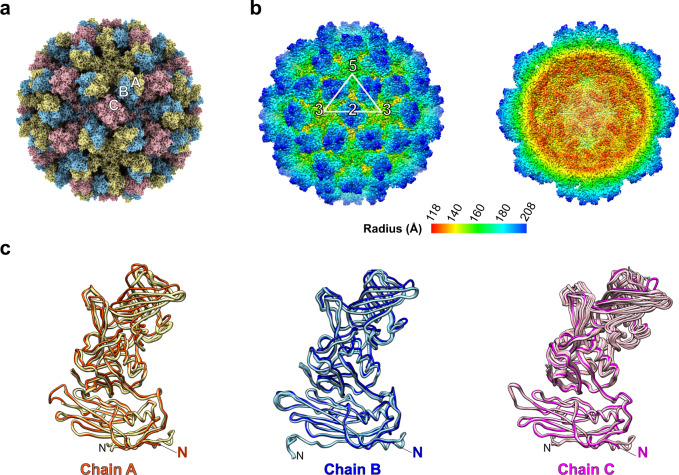
Cryo-EM structure of GII.4 HOV VLP. **a** GII.4 HOV VLP cryo-EM structure aligned to the crystal structure shown in Fig. 1 and viewed along the icosahedral twofold axis, shows the T = 3 symmetry of GII.4 HOV VLP in solution. The subunits A, B, and C are colored yellow, blue, and pink. **b** The surface and cut-away views of a GII.4 cryo-EM map colored by radial distance, showing similar particle radius as observed in the crystal structure. **c** Superimposition of subunit A (orange) with B (blue) or C (magenta) subunits of the cryo-EM structure with that of the VLP crystal structure (subunit A, yellow; subunit B, light blue; subunit C, pink) using the S domain. The N-terminus of VP1 in the cryo-EM and crystal structure is labeled with colored and black “N“s.

To investigate if the conformational flexibility observed in the crystal structure is indeed observed in solution and if the GII.4 VLP can conform to strict T = 3 icosahedrally symmetric organization, we determined the cryo-EM structure using a single-particle reconstruction method by imposing the icosahedral symmetry using cryo-SPARC (Supplementary Table 3). The cryo-EM images of the GII.4 VLP showed that the majority of particles were of the same diameter of ~400 Å. The final resolution of the cryo-EM structure is ~3.8 Å as estimated by Fourier Shell Correlation (FSC) of 0.143 (Supplementary Fig. 2a). Our cryo-EM analysis shows that GII.4 VLPs in solution also maintain the T = 3 capsid organization consisting of 180 VP1 subunits with outer and inner diameters of ∼410 Å and ∼230 Å, respectively (Fig. 3a, b). The quality of the 3.8 Å cryo-EM map allowed the unambiguous fitting of all the three quasi-equivalent VP1 subunits in the T = 3 icosahedral capsid, which showed well-defined densities for their S and P domains (Supplementary Figs. 2b, c). If, as in the crystal structure, the orientation of the P subunit with respect to the S domain (particularly in the C subunit) showed variations, the density would not have been as well defined because of icosahedral averaging. This indicates that the C subunit adopts a singular conformation, similar to one of the conformations in the crystal structure (Fig. 3c), to conform to the icosahedral symmetry. The conformations of the A and B subunits in the cryo-EM structure were the same as in the crystal structure. In all the three quasi-equivalent subunits, the P domain rests on the S domain in the resting conformation, as observed in the crystal structure (Fig. 3a). This is also evident in the cut-away view of the cryo-EM map, which shows continuous density between the S domain and the P domain (Fig. 3b). In contrast to the crystal structure, the densities for the N-terminal 43 residues of NTA were not observed in the cryo-EM map, perhaps because of the lower resolution of the cryo-EM map or the flexibility of the NTA in solution (Fig. 3c).

### Molecular interactions within and between VP1 subunits

**Fig. 4:**
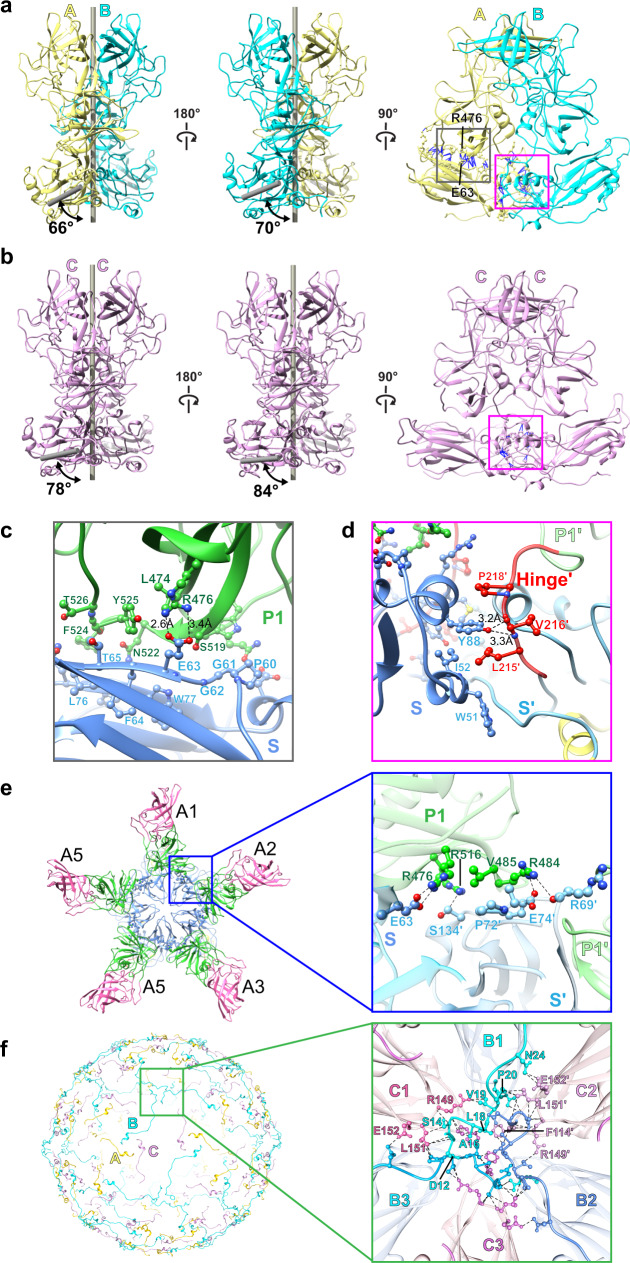
Molecular interactions within and between VP1 subunits. **a**, **b** Representative A/B and C/C dimer of the crystal structure. The angles formed by the P dimer and S domain axes are measured using Chimera, showing the bent A/B dimer with smaller angles and the flat C/C dimers with larger angles. The molecular interactions with and between VP1 are determined using Chimera and shown as blue lines. The S-P1 and S-Hinge interactions are indicated by gray and magenta boxes, respectively. **c** Close-up view showing the interactions between the P1 subdomain (green) and the S domain (blue). The interacting residues are shown as ball-and-stick models. **d** Close-up view of the interactions between the S domain (blue) and the hinge region of the neighboring subunit (red). The residues from the adjacent subunit are indicated with prime (′). **e** Interactions between the A1–A5 subunits at a five-fold axis. The inset shows the P1 subdomain of A1 interacting with the S domain of the A2 subunit. **f** The NTA network. Only residues of NTA are shown on the left panel for clarity. The inset presents how NTAs interact with each other and the S domains at a three-fold axis. The subunits B and C are colored in blue and pink, respectively.

Further analysis of the X-ray structure, in which the conformational details are resolved to a higher resolution, showed that the A, B, and C subunits exhibit noticeable differences in their intra-domain interactions (Fig. 4). In the A subunit, the S and P domains are closely associated via extensive molecular interactions, resulting in a more compact structure (Fig. 4a and c). The S domain residues, such as P60-T65 and L76, interact with the residues L474-R476 and S519-T526 of the P1 subdomain. The acidic residue E63 forms two salt bridges with the basic residue R476 (Fig. 4c). Whereas, in the B subunit, such S and P interactions are somewhat less, resulting in a relatively more flexible structure. Compared to the A and B subunits, the C subunit assumes the most open conformation, with no molecular contacts between the S and P domains (Fig. 4b). These differences contribute to the obligatory ‘bent’ conformation of the A/B dimers, which surround the icosahedral 5-fold axes, and the ‘flat’ conformation of the C/C dimers, at the icosahedral 2-fold axes, respectively (Fig. 4a, b). Such bent and flat conformations are a common feature in all the T = 3 icosahedral structures and are required for imparting the necessary curvature for the formation of the T = 3 capsid structure.

Despite the differences in the S-P1 interactions between A, B, and C subunits, the interactions involving the S domain with the hinge and the neighboring residues are common in all these subunits (Fig. 4a, b, and d). The hydrophobic residues W51, I52, and Y88 of the S domain closely associate with the hydrophobic residues L215, V216, and P218 of the hinge (Fig. 4d). In addition to interacting with the S domain of the same subunit, the P1 subdomain is also involved in the interaction with the S domain of the neighboring subunit. For instance, residue R484 interacts with E74′ and R69′ at the fivefold axis (Fig. 4e). These interactions contribute to the stability of the T = 3 capsid architecture.

### NTA ties the subunits through a network of interactions

One of the distinctive features of our crystal structure of the GII.4 VLP, compared to the previous cryo-EM structures of other GII.4 variants, is the detailed visualization of the extensive interactions mediated by the NTA (residues 1–43) that contribute to the stability of the capsid (Fig. 4f). In the crystal structure, the NTA of the B subunits, which is structurally more ordered than those of the A and C subunits, latches onto the neighboring C subunits via a network of molecular interactions (Fig. 4f). The residues D12-N24 of the B subunits surrounding the icosahedral 5-fold axes interact with the residues R149, L151, and E152 of the S domain of subunit C at the local 3-fold axes to form an extensive network of interactions. In the NTAs of the A and C subunits, the corresponding residues, D12-N24, are disordered. Interestingly, similar interactions involving the NTA of the B subunits are observed in the crystal structure of GI.1 Norwalk VLP^13^.

### The metal ion at the dimeric interface of the P domain

**Fig. 5:**
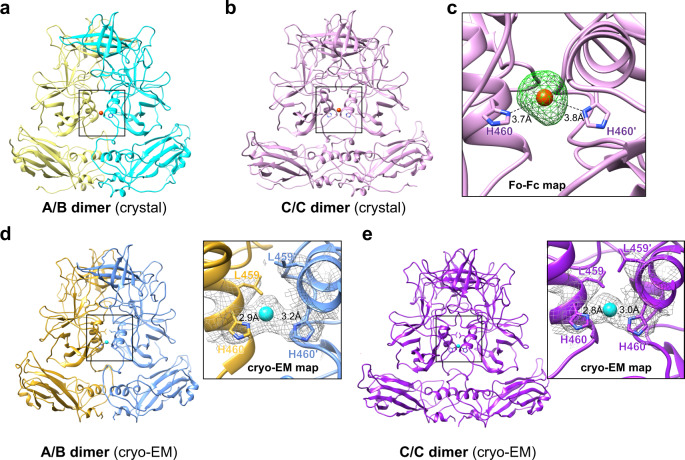
Ion binding site at the dimeric interface of VP1. **a**, **b** Cd^2+^ metal ion (orange sphere) is bound between the P dimers via a conserved residue His460. **c** Simulated annealing omit map of the crystal structure is shown as green mesh and contoured at 3σ level. **d**, **e** Cryo-EM density maps of A/B and C/C dimers reveal density (gray mesh) at the same site. The side chains of Leu459 and His460 are shown as stick models. A water molecule is placed into the density between these Histidine residues.

Another novel finding in our crystallographic structure of the GII.4 VLP is the strongly resolved electron density in the unbiased simulated annealing omit map. This density is present between the P1 subdomains in all the VP1 dimers in the crystallographic asymmetric units. Because the crystals of GII.4 VLP were obtained in a condition containing 100 mM cadmium chloride, we interpreted this strong density as possibly due to a Cd^2+^ ion (Fig. 5a–c). The simulated annealing omit map contoured at 3 σ level is consistent with a Cd^2+^ ion coordinated by the residue His460 at the dimeric interface of P1 subdomains (Fig. 5c). In the protein crystals, histidine is often involved in coordinating various metal ions, including cadmium^28^. A similar strong density is also present at the same location in our 3.8 Å cryo-EM density map (Fig. 5d, e), suggesting an intrinsic affinity for a divalent cation at this position of the capsid protein.

### Removal of ion affects the capsid structure

**Fig. 6:**
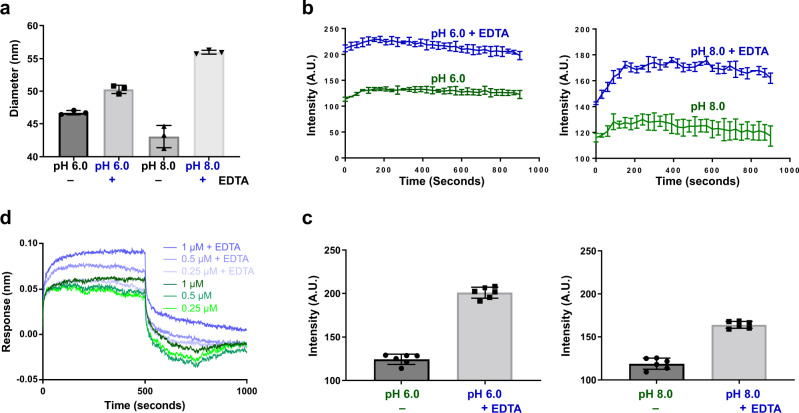
Dynamic light scattering, Bis‐ANS, and BLI binding assays. **a** DLS analysis shows the hydrodynamic radius increase upon removal of metal ions by chelation with EDTA at pH 6.0 and pH 8.0, indicating the rising of P domain above shell in the absence of ion at the dimeric interface. **b** Changes in bis-ANS binding in the absence or presence of EDTA show increased fluorescence intensity when VLPs are incubated with 20 mM EDTA, suggesting more hydrophobic surfaces of VLP bound with bis-ANS. **c** Stabilized fluorescence intensities measured during the last minute for each sample were averaged and presented as a bar graph. **d** BLI analysis of NORO-320 Fab and GII.4 HOV VLP shows that more VLPs bind to the immobilized Fab of GII.4 mAb NORO-320 in the presence of 20 mM EDTA, suggesting the exposure of mAb-binding epitope with EDTA-treatment. Data presented in each panel are means ± SE (*n* = 3 independent study repliates) are shown. Source data are provided.

To investigate if the removal of the ion had any effect on the GII.4 capsid, we first performed dynamic light scattering (DLS) in the absence and presence of the chelating agent EDTA (Fig. 6a). Remarkably, the DLS analysis indicated a monodisperse population of VLPs with a significant increase in the hydrodynamic radius in the presence of EDTA at pH 6.0 and pH 8.0 (Fig. 6a). We then performed a bis-ANS binding assay in the presence and absence of EDTA to examine if loss of ion binding exposed new hydrophobic surfaces. This assay showed that indeed is the case as there was a substantial increase in the fluorescence intensity in the presence of EDTA (Fig. 6b, c). To further investigate if EDTA treatment of the HOV VLP had any effect on the antigen presentation and antibody binding, we performed BLI experiments using biotinylated Fab of a neutralizing antibody NORO-320 that binds to the sides of the P domain dimer^29^. These experiments showed that EDTA treatment increases the binding of NORO-320 Fab to the VLP, indicating that chelation of the metal ion results in a conformational change that allows for increased exposure of the partially occluded epitope for Fab binding (Fig. 6d).

In addition to the above studies, we analyzed the cryo-EM images of the HOV VLP treated with EDTA to examine if the particles showed a discrete or a continuous heterogeneity using the recently developed three-dimensional variability analysis (3DVA) in cryoSPARC^30^. This analysis shows that upon EDTA treatment, the particle images split into two populations (Supplementary Fig. 5) in clear contrast to untreated VLPs which exhibit a homogeneous single distribution of VLP images (Supplementary Fig. 5a). The visualization of the particle variability in 3D shows that EDTA treatment leads to increased particle diameter with thinning of the density between the P and S domains indicating a transition from resting to a raised conformation of the VP1 (Supplementary Movies 1 and 2). Taken together, these experiments suggest that loss of ion binding has a profound effect on the capsid structure leading to a raised conformation with increased access to antigenic sites.

## Discussion

In this study, by determining the structure of a globally dominant GII.4 HuNoV using X-ray crystallography and cryo-EM, we have provided the atomic details of how the major capsid protein VP1 assembles into a native T = 3 icosahedral assembly (Figs. 1 and 3). Although this is the first crystallographic structure of the GII.4 VLP, previously, cryo-EM structures of VLPs of two GII.4 variants have been reported^12,14,20^. These cryo-EM structures showed unique aspects of the GII.4 capsid structure that were not observed in our GII.4 structure. In the 4.1 Å cryo-EM structure of the VLP of GII.4 Minerva strain, the capsid displays T = 4 icosahedral organization, which had not been observed before in the members of *Caliciviridae*^14^. In the 8.1 Å cryo-EM structure of the GII.4 VLP expressed to mimic a vaccine strain, the capsid exhibits a T = 3 symmetry^12^, although the VP1 subunits, as in the T = 4 structure of the Minerva strain, exhibit a ‘rising’ conformation, which was not observed previously except in the case of murine NoV. Our studies by providing the structural details at the atomic level have allowed insights into the factors that likely influence the conformational dynamics of GII.4 VP1 and how it could impact the symmetry and stability of the capsid.

### The VP1 flexibility observed in GII.4 capsid is likely an intrinsic feature of HuNoV capsids

A remarkable observation in our 3.0 Å crystal structure of the GII.4 VLP is the extent to which the conformation of capsid protein can deviate within the context of an icosahedrally symmetric organization. This observation was possible because the GII.4 VLP crystallized in the space group I222, with 45 VP1 subunits in the crystallographic asymmetric unit representing 15 of each of the three quasiequivalent subunits in the T = 3 icosahedral lattice (Supplementary Fig. 1). Our structure determination without imposing icosahedral symmetry showed that the C subunits varied significantly in their conformations compared to the A and B subunits. The deviations are not in the conformation of the individual domains, which remain mostly invariant between the subunits, but are due to distinct conformational variations in the hinge region, allowing the P domain in these subunits to be oriented differently with respect to the S domain (Fig. 2c). These variations in the hinge are mostly due to the significant changes in the backbone conformational angles of the residue T219 following two proline residues in the hinge region. Compared to T219-E221, other residues (213–218) in the hinge region show minor variations. High conservation of the residues in the hinge region of the VP1 suggests similar conformational variability is likely an intrinsic feature in the capsids of other HuNoVs.

Interestingly, such variations are not observed in the cryo-EM structure. Despite enforcing icosahedral symmetry, the density for the three quasi-equivalent subunits is well defined. The conformations of the A and B subunits are the same as in the X-ray structure, whereas the C subunit is ‘locked’ into a singular conformation selected from one of the 15 conformations observed in the crystal structure to adhere to the icosahedrally symmetric organization. These observations indicate that under the influence of external forces such as crystal packing, in this case the capsid protein has certain conformational freedom to ‘breathe’ because of the flexible hinge. It is plausible that such intrinsic conformational flexibility plays a role in optimal engagement with cellular receptors upon contacting the cell surface during cell entry processes.

### From ‘resting’ to ‘raised’ conformation of VP1

In both of our crystal and cryo-EM structures of the GII.4 VLP, the P domains of all the three quasi-equivalent subunits remain close to the S domain in a ‘resting’ conformation, similar to that observed in the high-resolution T = 3 structures of the several animal caliciviruses and the other HuNoV VLPs. However, in the two recent cryo-EM structures of GII.4 VLPs, irrespective of T = 3 or T = 4 organization, as noted above, all the three quasiequivalent VP1 subunits are in the ‘raised’ conformation with the P domain rotated and significantly elevated from the S domain^12^. Such a raised conformation of the VP1 was first observed in the 8.1 Å cryo-EM structure of the murine norovirus (MNV) with T = 3 symmetry^31^. Follow-up cryo-EM studies with MNV VLPs showed VP1 transits to the resting state in the presence of bile acids and that the resting state promotes efficient interactions with the cellular receptor^23^. However, in the most recent 3.1 Å cryo-EM structure of the MNV, the capsid protein is in a resting state both in the absence and presence of bile acids^22^. Nonetheless, these structural studies show that the P domain of the norovirus capsid protein, because of the flexible hinge, can transit from a resting to a rising state. Such a transition appears to depend upon several conditions.

### Factors that favor ‘resting’ vs. ‘raised’ states of VP1

**Fig. 7:**
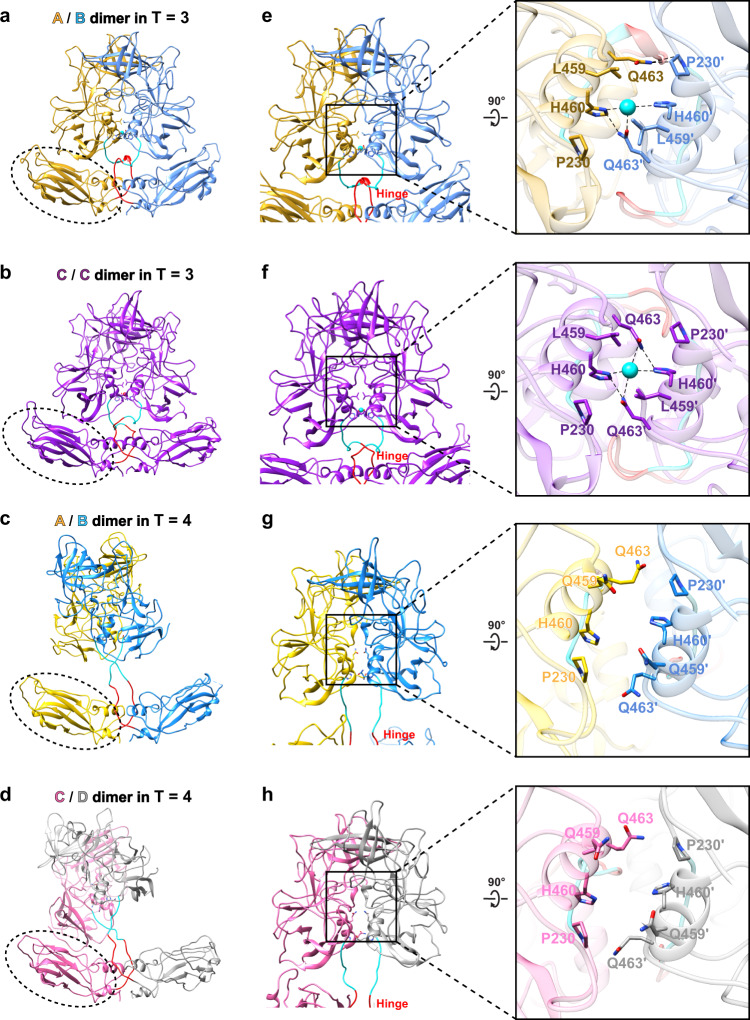
Comparison of different quasiequivalent VP1 dimers in the resting conformation in T = 3 HOV capsid vs the raised conformation in T = 4 Minerva capsid. Comparison of the dimers using the S domain (shown in dashed oval) (left panel, **a**–**d**), or the P domain (middle panel, **e**–**h**) for structural alignment. The additional C/D dimer present in the T = 4 capsid is shown in the bottom row (**d, h**). The inset (right panel) corresponding the the boxed region in the middle panel shows a close up view of the interactions at the dimeric interface viewed along the two-fold axis of the P dimer. The ion present only in the T = 3 dimers is shown as a cyan sphere. The A, B, C, and D subunits are labeled, and colored in yellow, blue, purple, and gray respectively (the C subunit of the T = 4 C/D dimer is shown in lighter shade of purple to distinguish it from the C subunits of C/C dimer)). Residues 213-221 (hinge) and 222-229 (N-terminus of P domain) are colored red and cyan, respectively.

Comparison of the resting conformation of VP1 as found in our structural studies with that of the ‘raised’ conformation of the VP1 as observed in the recent 4.1 Å cryo-EM structure of the Minerva GII.4 VLP, we notice several interesting differences despite the overall structures of the S and the P domain remaining highly similar with a RMSD of ~2.23–2.31 Å and ~2.15–2.30 Å, respectively, when the individual S or P domains are compared alone (Fig. 7). The first difference is the loss of all interactions between the S and P domain in the ‘raised’ conformation as the P domain, with the hinge region fully extended, is rotated and elevated from the S domain by as much as 24 Å (Fig. 7c, d). The second difference is the presence of metal ions at the P domain dimeric interface in our structures, which is absent in the ‘raised’ conformation. The residues at the dimeric interface that are close to the metal ion in the resting conformation move away in the rising conformation creating a gap at the dimeric interface (Fig. 7g, h and Supplementary Fig. 4). With the absence of S-P interactions and the metal ion, it appears that the capsid structure with the ‘raised’ conformation of the VP1 subunits would be more labile and less stable compared to the capsid with the subunits in the resting conformation. Furthermore, in the resting state, the interactions between the P and S domains are considerable, with a buried surface area of 2078 Å^2^, as seen by analyzing the interactions between S (residues 1–213) of the chain with the rest of the protein using the program PISA^32^. The calculated ΔG, binding energy, from the same program, is −8.6 kcal/mol. In the raised state, these stabilizing interactions are lost.

Although the metal ion observed in our crystal structure is likely Cd^2+^, based on the composition of the crystallization buffer, the nature of the metal ion in the cryo-EM density is uncertain. Postulating that it is likely a divalent metal ion, we examined the effect of treating the GII.4 VLPs with EDTA using DLS and Bis-ANS assay (Fig. 6). These experiments show that EDTA treatment results in a noticeable increase in the VLP diameter as well as exposure of new hydrophobic surfaces, suggesting that the chelation of the metal ion triggers a possible transition from a resting conformation to a raised conformation as noticed using 3DVA analysis. Interestingly, the EDTA treatment coupled with an increased pH, as inferred from the ~8.1 Å resolution cryo-EM studies, has been shown to trigger such a transition in MNV and as well as in the T = 3 capsid structure of HuNoV GII.3 VLP^21^. The authors further showed that transition between resting and rising conformations is reversible both in MNV and GII.3 VLP and that the infectivity of MNV is optimal under the conditions favoring the resting conformation.

**Fig. 8:**
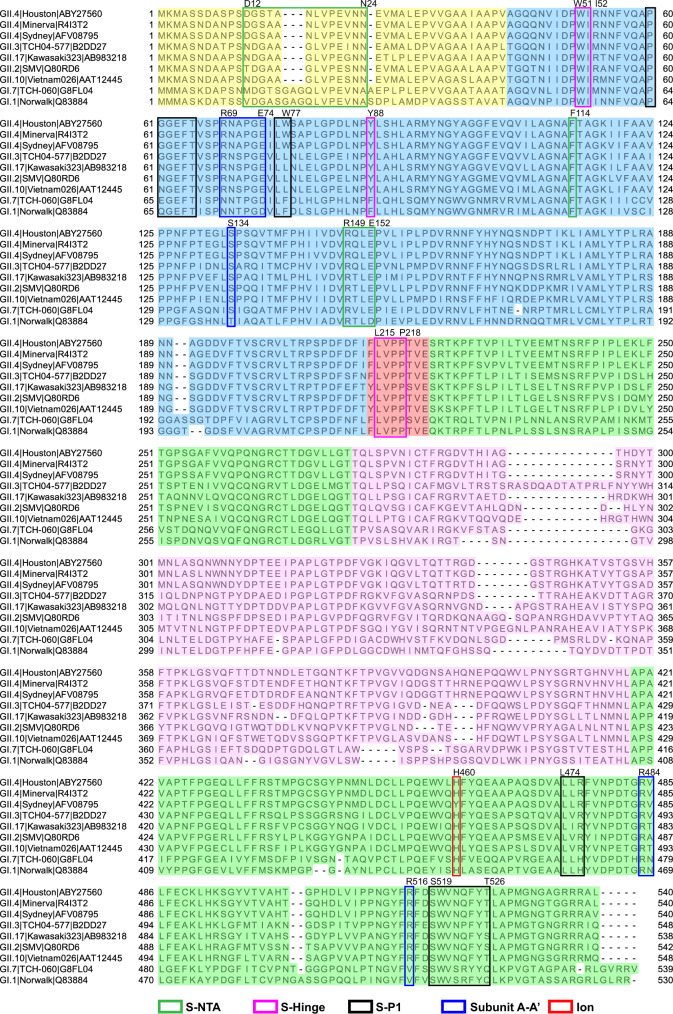
Sequence alignment of HuNoV VP1. Multiple sequence alignment was carried using ClustalW and visualized by Jalview^50^. The genotype | strain | accession number of each VP1 sequence is given on the left side of the alignment. Residues are color-shaded, corresponding to the schematic in Fig. 1a. The residues involved in the inter-domain or inter-subunit interactions are indicated using colored boxes.

Our structure suggests a possible mechanism of how the transition from a resting conformation can occur in the GII.4 capsid. As observed in our GII.4 VLP structure, the metal ion binding site is close to the hinge region. The removal of the bound metal ion may allosterically affect the conformation of the hinge allowing the P domain to transit from a resting to a rising state. The pairs of H460, Q463, L459 from the opposing subunits in the P dimer are involved in metal ion binding. Sequence comparisons show that these residues are well conserved in HuNoV VP1 sequences, including GII.3 (Fig. 8). However, interestingly, in GII.4 Sydney (2012), H460 is replaced by Y460, and a hydrophilic residue Q459 replaces L459 in GII.4 Minerva and GII.4 Sydney (Fig. 8). Whether these sequence changes alter the metal binding affinity and tilt the preference for either the resting or the raised state, or whether they alter the transition kinetics remains unclear.

### Possible implications of ‘resting’ to ‘raised’ transition in antibody neutralization

**Fig. 9:**
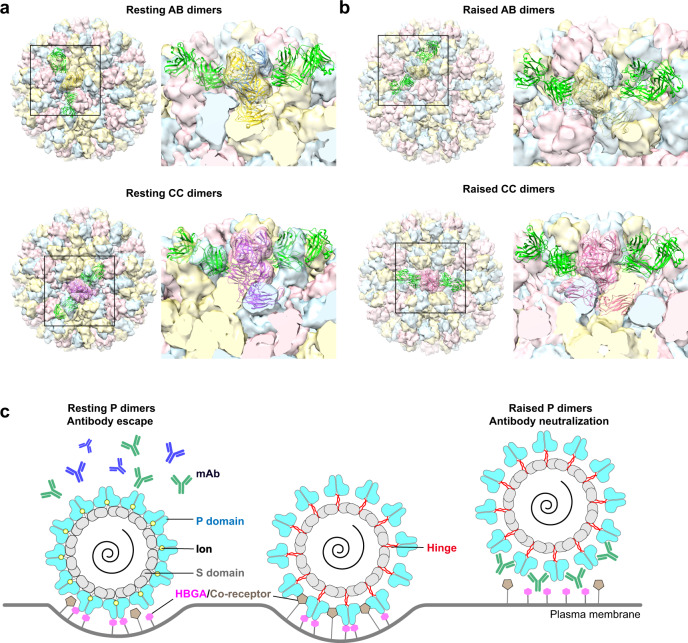
Model of GII.4 HOV VLP in the ‘resting’ and ‘raised’ conformations. **a** Superimposition of GII.4 P domain in complex with Fab of NORO-320 onto the AB and CC dimers of HOV VLP cryo-EM structure at 3.8 Å. The neighboring VP1 is shown as a transparent surface representation. **b** Modeling of GII.4 HOV VLP in the ‘raised’ conformation using the 8.0 Å GII.4c cryo-EM map in T = 3 symmetry. The P domain/Fab320 complex is superimposed onto the model as in (**a**). **c** Schematic of NoV cell attachment process and human mAb neutralization mechanism.

Our GII.4 structure also supports the suggestion by Song et al. that the transition from a more stable resting conformation to a more dynamic ‘raised’ conformation of VP1 may play a critical role during the cell entry processes or the disassembly processes. In addition to the cryo-EM structures of MNV and various HuNoVs VLPs, which have captured the resting and the raised states individually, a clear demonstration by Song et al., that such a transformation is reversible strongly suggests that this dynamic aspect is intrinsic to norovirus VP1. Dynamic structural transitions have been implicated in the cell entry process for several viruses including, for example, the spike proteins of rotavirus, influenza virus, and coronavirus^18,33–36^. If such a transition is indeed a critical step in the downstream cell entry events of HuNoV, it also may have a consequence in how specific antibodies recognize and neutralize the virus infection as some of the antigenic sites may become more accessible in one of these conformations. This appears the case with the human antibody NORO-320 that efficiently neutralizes GII.4 and GII.17 HuNoV infections in the human enteriod system^29^. As determined by the crystal structure of the GII.4 P domain in complex with the antigenic binding fragment (Fab) of this antibody, the antigenic site is on the sides of the P domain at a region that is conserved in the GII genotypes. Mapping this site on our GII.4 capsid structure, with the subunits in the resting conformation, indicates that this site is occluded to a large extent, limiting the antibody access for efficient binding and neutralization (Fig. 9a). However, when we model the ‘raised’ conformation of the P domain into our structure, the NORO-320 binding site is fully exposed. It allows a stoichiometrically higher number of antibodies to bind and thus increasing the neutralization efficiency (Fig. 9b and Supplementary Fig. 3). Consistent with this hypothesis, the BLI assay shows increased binding of NORO-320 to VLPs in the presence of EDTA (Fig. 6d). Similarly, the antigenic site for one of the nanobodies, although whether it neutralizes virus infection is not determined, becomes more accessible in the ‘raised’ conformation^37^. However, such a transition may not be of consequence for antibodies that neutralize HuNoV infection by blocking the HBGA binding site as these sites are not affected by the transition and remain accessible in both conformations (Fig. 9c).

In summary, we have provided the first atomic-level description of the capsid architecture of a globally dominant GII.4 HuNoV in the native T = 3 icosahedral symmetric state. In both the crystal and solution, the capsid protein VP1 assumes a resting state stabilized by an elaborate network of NTA linking the capsid subunits and the interactions between the S and P domains, as expected in a T = 3 icosahedral capsid. A novel finding is the metal ion binding at the dimeric interface of the P domain that further stabilizes the resting conformation. A remarkable feature in our crystal structure is how the flexible hinge allows the metal ion stabilized P domain to sample an extensive conformational space in the resting conformation and allows transition to possibly a more extended rising conformation with the removal of the metal ion. Such conformational plasticity perhaps is an inherent feature of the norovirus VP1 akin to the spike proteins of many enveloped and nonenveloped viruses, and maybe a critical factor in optimizing the interactions with the receptor and subsequent penetration through the cell membrane and genome release. It is hoped that such a detailed understanding of the GII.4 capsid architecture will provide a rational basis for the improved design and development of VLP-based vaccines to counter global HuNoV infections.

## Methods

### Expression and purification of GII.4 HOV VLP

The VP1 and VP2 of a GII.4 norovirus (Houston/TCH186/2002 or HOV, GenBank no. EU310927) were expressed in the baculovirus system using Gibco Sf9 cells as described previously^13^. Briefly, cells were grown for 10 days and then centrifuged for 15 min at 1120 × g. The supernatant containing VLPs was then clarified by centrifugation at 22,100 × g for 30 min to remove aggregates and cell debris. Next, the clarified supernatant was transferred to a fresh tube, and a 30% sucrose cushion was gently added to the bottom of the tube. This sample containing the 30% cushion was then centrifuged at 120,000 × g for 3 h to pellet the VLPs. VLPs were then suspended by adding 200 uL of PBS per tube and incubating at 4 °C overnight. Suspended VLPs were then pooled and diluted with PBS containing cesium chloride (1.14 mg/ml) to a final concentration of 0.38 mg/ml cesium chloride. The sample was then centrifuged at 150,000 × g for 48 h at 4 °C. Lipid layer was removed, and then a visible blue band containing VLP was isolated from each gradient tube. The VLP sample was then dialyzed overnight at 4 °C in PBS pH6.0. Dialyzed VLPs were then further purified using the Sephacryl S500 size exclusion chromatography column. Purified fractions were then pooled and stored at 4 °C.

### Crystallization of GII.4 HOV VLP

Crystallization trials were carried out using the hanging-drop vapor diffusion method at room temperature. For each condition, 2 μl of the VLP solution, at a concentration of 3 mg/ml, was mixed with 2 μl of the well solution and equilibrated with 0.5 ml of the well solution. Initial screening produced crystals under a few conditions, including the Crystal Screen II condition 12 (0.1 M Na acetate pH 4.6, 0.1 M cadmium chloride, 30% PEG 400). After crystallization condition optimization and on-site synchrotron screening, crystals of the best diffraction quality were obtained using a well solution containing 0.1 M Na acetate pH 3.6, 26–27% PEG 400. Cryo-protection of crystals was obtained by soaking the crystals in the mother liquor with increasing glycerol concentration through four steps: 5%, 10%, 15%, and 18%. The equilibration time at each solution was at least 10 min. The crystals were then flash-frozen in liquid nitrogen and shipped to synchrotron sites for data collection.

### Crystal structure data collection and analysis

The diffraction data from single crystals were collected under cryo-conditions at APS 19-ID with monochromatic X-rays (wavelength = 0.9795 Å) and a detector to crystal distance of 350 mm on an ADSC 3 × 3 CCD detector using an oscillation angle of 0.3° and an exposure time of 5 sec. Indexing, integration, scaling, post-refinement, and data reduction were carried out using the HKL2000^38^. Analysis of the diffraction data indicated that the HOV VLP crystal belonged to the orthorhombic space group I222 (Supplementary Table 2). The data between 30 and 3.0 Å from 300 frames were scaled with a Rmerge factor of about 13%. To determine the precise orientation of the virus particle in the unit cell, self-rotation functions were calculated using the program GLRF^39^. These calculations showed all the peaks corresponding to 5-fold (κ = 72°), 3-fold (κ = 120°), and 2-fold (κ = 180°) axes, which are expected from a particle with icosahedral symmetry. In the I222 space group, consistent with the unit cell dimensions and the particle radius, which is estimated to be 200 Å, the particle position is uniquely defined by the intersection of the crystallographic 2-fold axes. In such a setting, an icosahedral particle can assume one of the two possible orientations. This ambiguity in orientation was resolved by the self-rotation function calculations using the X-ray diffraction data. Each crystallographic asymmetric unit is composed of 1/4th of the virion with a non-crystallographic 15-fold symmetry. Similar packing of icosahedral particles has been observed in the Norwalk virus.

### Crystal structure determination and refinement

A properly positioned and oriented Norwalk capsid was placed in an HOV crystal unit cell. One initial model at 10 Å was calculated from the 15 copies of NV related first by icosahedral 5-fold symmetry and then by icosahedral 3-fold symmetry. Another initial model was obtained from a cryo-EM reconstruction of HOV at a resolution of 22 Å. The cryo-EM structure was also used to generate a mask. Phase refinement and extension were carried out by iterative cycles of real space electron density averaging, solvent flattening, and back transformation with CCP4 program packages^40^. The phases were extended to the final 3 Å resolution, with each step less than one reciprocal space point. The averaged 3.0 Å density map is of good quality and readily interpretable in the whole S domain and the P domains of the A and B subunits, but not in the P domain of the C subunit. Iterative cycles of refinement and further model building were carried out using PHENIX^41^ and COOT programs^42^. Non-crystallographic symmetry (NCS) was applied during refinement using Phenix.refine. In the final refinement, the stereochemistry of the structures was checked using MolProbity^43^. Data refinement and statistics are given in Supplementary Table 2. The interactions within and between VP1 subunits were analyzed using Chimera^44^. Figures were prepared using Chimera and ChimeraX^44,45^.

### Cryo-EM sample preparation and data collection

A 3.5 μL aliquot of GII.4 HOV VLPs (0.24 mg/ml) in a buffer containing PBS pH 6.0 was applied onto a 200-mesh R2/1 Quantifoil holey carbon grid coated with 0.2 mg/ml Graphene Oxide. The grid was blotted and rapidly frozen in liquid ethane using a Vitrobot IV(FEI), with constant temperature and humidity during the process of blotting. Movie stacks (3149) were collected at 300 kV on a JEM-3200FSC electron microscope (JEOL) with an in-column energy filter (50-eV width) equipped with a direct electron detector K2 Summit camera (Gatan). Images were collected semiautomatically by SerialEM^46^ in dose fractionation super-resolution counting mode with a binning of 0.5 at 30,000X magnification, corresponding to a pixel size of 1.2 Å. The images were collected with a defocus range from −0.6 to −2.6 μm. The final frame average was computed from 25 frames to correct for beam-induced motion during exposure by MotionCor2^47^. The image in each frame was weighted according to radiation damage. CTF parameters of the particles in each frame average were determined by Relion 3.1 CtfFind and Gctf v1.06^48^. Two-dimensional (2D) reference-free class averages were computed using RELION 3.1. Data were processed for icosahedral reconstruction using CryoSPARC. The 0.143 Fourier shell correlation (FSC) cut-off was used for the resolution determination (Supplementary Fig. 2). Three-dimensional variability analysis (3DVA) implemented in cryoSPARC^30^ was performed to understand and visualize the dynamics of GII.4 HOV VLPs (0.24 mg/ml) in a buffer containing PBS pH 6.0 in the presence of 20 mM EDTA (Supplementary Movies 1 and 2).

### Cryo-EM structure determination and refinement

The crystal structure of GII.4 HOV VLP was fitted into the density map using Chimera. Iterative cycles of refinement and further model building were carried out using PHENIX^41^, Rosetta^49^, and COOT programs^42^ Water molecules were built using Douse in PHENIX and manually verified in COOT. The cryo-EM map was deposited in the Electron Microscopy Databank, and the coordinates of the atomic model were deposited in the Protein Data Bank. EM density Data collection and processing details are provided in Supplementary Table 3.

### Dynamic light scattering

The hydrodynamic diameters of GII4 HOV VLPs in the absence or presence of the ion chelation reagent EDTA at pH 6.0 or pH 8.0 were measured using dynamic light scattering (DLS) on a ZetaSizer Nano instrument (Malvern Instruments, UK). Samples were diluted to a final concentration of 200 nM for each component in phosphate-buffered saline. Upon addition of EDTA, samples were incubated on ice for 30 min before measurements were taken. Three × 12 measurement runs were performed with standard settings (Refractive Index 1.335, viscosity 0.9, temperature 25 °C). The average result was created with ZetaSizer software.

### Detection of bis-ANS binding by fluorescence spectroscopy

Purified VLPs (30 μg/ml, 0.5 μM concentration of the VP1) diluted in PBS buffer pH 6.0 or pH 8.0 with or without 20 mM EDTA were incubated at 25 °C for 10 min to allow for temperature equilibration. To detect bis-(8-anilinonaphthalene-1-sulfonate) (bis-ANS) binding to VLP alone or in the presence of EDTA, bis-ANS was added to each sample for a final concentration of 3 μM immediately before data collection. Bis-ANS was excited at 395 nm, and emission was collected at 495 nm at 30 s intervals for 15 min on a Flexstation 3 (Molecular Devices, USA). The averaged results were prepared using PRISM.

### Modeling of GII.4 HOV VLP in the ‘raised’ conformation

The atomic model of GII.4 VLP P domain and S domain were fit into the 8.1 Å cryo-EM density map of norovirus GII.4c VLPs with T = 3 icosahedral symmetry using Chimera. The flexible hinge was built using COOT. The axis of P domain dimers was defined using mass-weighting in Chimera. We then calculated the crossing angle and distance of the axes of the P domain in the ‘rising’ and ‘raised’ conformation using Chimera.

### Biolayer interferometry (BLI)

Biotinylation of the NORO-320 Fab was carried out using EZ-Link NHC-LC-LC-biotin (catalog no. 21343; Thermo Scientific) following the manufacturer’s instructions. The NORO-320 Fab was loaded onto streptavidin biosensors at a concentration of 1 μg/ml in the BLI running buffer (20 mM Hepes, 150 mM NaCl, 0.005% surfactant P20, and 2 mg/ml BSA, pH 7.8) for 600 s, resulting in capture levels of 1.5–2 nm within a row of eight tips. GII.4 HOV VLP was diluted in BLI running buffer with or without the addition of 20 mM EDTA to a final concentration of 1000 nM and incubated on ice overnight. NORO-320 Fab-GII.4 HOV VLP association and dissociation curves were obtained through twofold serial dilutions of GII.4 HOV VLP (1000, 500, 250 nM) plus buffer blanks at 30 °C using the Octet acquisition software. BLI studies were carried out using an Octet RED96 instrument (FortéBio).

### Reporting summary

Further information on research design is available in the Nature Research Reporting Summary linked to this article.

## Supplementary information

Supplementary Information

Peer Review File

Description of Additional Supplementary Files

Supplementary Movie 1

Supplementary Movie 2

Reporting Summary

## Data Availability

The data that support this study are available from the corresponding author upon reasonable request. Atomic coordinates and structure factors for the crystal structure of the GII.4 HOV VLP have been deposited in the Protein Data Bank (PDB) with the accession code 7K6V [10.2210/pdb7K6V/pdb]. The cryo-EM structure of GII.4 HOV VLP has been deposited in the Protein Data Bank with the accession code 7MRY [10.2210/pdb7MRY/pdb] and the Electron Microscopy Databank with accession code EMD-23960. Source data are provided with this paper.

## Source data

Source data

## References

[1] Villabruna, N., Koopmans, M. P. G. & de Graaf, M. Animals as reservoir for human norovirus. *Viruses* **11**,10.3390/v11050478 (2019). 10.3390/v11050478 PMC6563253 31130647

[2] Mattison CP, Cardemil CV, Hall AJ (2018). Progress on norovirus vaccine research: public health considerations and future directions. Expert Rev. Vaccines.

[3] Kirk MD (2015). World Health Organization estimates of the global and regional disease burden of 22 foodborne bacterial, protozoal, and viral diseases, 2010: a data synthesis. PLoS Med.

[4] GBD 2015 Mortality and Causes of Death Collaborators. Global, regional, and national life expectancy, all-cause mortality, and cause-specific mortality for 249 causes of death, 1980–2015: a systematic analysis for the Global Burden of Disease Study 2015. *Lancet* **388**, 1459–1544 (2016). 10.1016/S0140-6736(16)31012-1 PMC5388903 27733281

[5] Vongpunsawad S, Venkataram Prasad BV, Estes MK (2013). Norwalk virus minor capsid protein VP2 associates within the VP1 shell domain. J. Virol..

[6] Chhabra P (2019). Updated classification of norovirus genogroups and genotypes. J. Gen. Virol..

[7] Sherwood J (2020). Efficacy of an intramuscular bivalent norovirus GI.1/GII.4 virus-like particle vaccine candidate in healthy US adults. Vaccine.

[8] Kim, L. et al. Safety and immunogenicity of an oral tablet norovirus vaccine, a phase I randomized, placebo-controlled trial. *JCI Insight* **3**, 10.1172/jci.insight.121077 (2018). 10.1172/jci.insight.121077 PMC6124525 29997294

[9] Siebenga JJ (2007). Epochal evolution of GGII.4 norovirus capsid proteins from 1995 to 2006. J. Virol..

[10] Ettayebi K (2016). Replication of human noroviruses in stem cell-derived human enteroids. Science.

[11] Ettayebi, K. et al. New insights and enhanced human norovirus cultivation in human intestinal enteroids. *mSphere* **6**, 10.1128/mSphere.01136-20 (2021). 10.1128/mSphere.01136-20 PMC7885322 33504663

[12] Devant JM, Hansman GS (2021). Structural heterogeneity of a human norovirus vaccine candidate. Virology.

[13] Prasad BV (1999). X-ray crystallographic structure of the Norwalk virus capsid. Science.

[14] Jung J (2019). High-resolution cryo-EM structures of outbreak strain human norovirus shells reveal size variations. Proc. Natl Acad. Sci. USA.

[15] Ossiboff RJ, Zhou Y, Lightfoot PJ, Prasad BV, Parker JS (2010). Conformational changes in the capsid of a calicivirus upon interaction with its functional receptor. J. Virol..

[16] Chen R, Neill JD, Estes MK, Prasad BV (2006). X-ray structure of a native calicivirus: structural insights into antigenic diversity and host specificity. Proc. Natl Acad. Sci. USA.

[17] Wang X (2013). Atomic model of rabbit hemorrhagic disease virus by cryo-electron microscopy and crystallography. PLoS Pathog..

[18] Conley MJ (2019). Calicivirus VP2 forms a portal-like assembly following receptor engagement. Nature.

[19] Prasad BV (2016). Antiviral targets of human noroviruses. Curr. Opin. Virol..

[20] Devant JM, Hofhaus G, Bhella D, Hansman GS (2019). Heterologous expression of human norovirus GII.4 VP1 leads to assembly of T = 4 virus-like particles. Antivir. Res.

[21] Song C (2020). Dynamic rotation of the protruding domain enhances the infectivity of norovirus. PLoS Pathog..

[22] Snowden JS (2020). Dynamics in the murine norovirus capsid revealed by high-resolution cryo-EM. PLoS Biol..

[23] Sherman, M. B. et al. Bile salts alter the mouse norovirus capsid conformation: possible implications for cell attachment and immune evasion. *J. Virol.* **93**, 10.1128/JVI.00970-19 (2019). 10.1128/JVI.00970-19 PMC6744230 31341042

[24] Taniguchi K, Urasawa S, Urasawa T (1981). Further studies of 35–40 nm virus-like particles associated with outbreaks of acute gastroenteritis. J. Med. Microbiol..

[25] Bertolotti-Ciarlet A, White LJ, Chen R, Prasad BV, Estes MK (2002). Structural requirements for the assembly of Norwalk virus-like particles. J. Virol..

[26] Sosnovtsev SV, Green KY (2000). Identification and genomic mapping of the ORF3 and VPg proteins in feline calicivirus virions. Virology.

[27] Glass PJ (2000). Norwalk virus open reading frame 3 encodes a minor structural protein. J. Virol..

[28] Dokmanic I, Sikic M, Tomic S (2008). Metals in proteins: correlation between the metal-ion type, coordination number and the amino-acid residues involved in the coordination. Acta Crystallogr D. Biol. Crystallogr.

[29] Alvarado G (2021). Broadly cross-reactive human antibodies that inhibit genogroup I and II noroviruses. Nat. Commun..

[30] Punjani A, Fleet DJ (2021). 3D variability analysis: resolving continuous flexibility and discrete heterogeneity from single particle cryo-EM. J. Struct. Biol..

[31] Katpally U (2010). High-resolution cryo-electron microscopy structures of murine norovirus 1 and rabbit hemorrhagic disease virus reveal marked flexibility in the receptor binding domains. J. Virol..

[32] Krissinel E, Henrick K (2007). Inference of macromolecular assemblies from crystalline state. J. Mol. Biol..

[33] Herrmann T (2021). Functional refolding of the penetration protein on a non-enveloped virus. Nature.

[34] Russell, C. J. Hemagglutinin stability and its impact on influenza A virus infectivity, pathogenicity, and transmissibility in avians, mice, swine, seals, ferrets, and humans. *Viruses* **13**, 10.3390/v13050746 (2021). 10.3390/v13050746 PMC8145662 33923198

[35] Wang, L. & Xiang, Y. Spike glycoprotein-mediated entry of SARS coronaviruses. *Viruses* **12**, 10.3390/v12111289 (2020). 10.3390/v12111289 PMC7696831 33187074

[36] Williams, A. N. et al. Multiple signals in the gut contract the mouse norovirus capsid to block antibody binding while enhancing receptor affinity. *J. Virol*. 10.1128/JVI.01471-21 (2021). 10.1128/JVI.01471-21 PMC8549501 34468172

[37] Koromyslova AD, Hansman GS (2017). Nanobodies targeting norovirus capsid reveal functional epitopes and potential mechanisms of neutralization. PLoS Pathog..

[38] Otwinowski Z, Minor W (1997). Processing of X-ray diffraction data collected in oscillation mode. Methods Enzymol..

[39] Tong L, Rossmann MG (1997). Rotation function calculations with GLRF program. Methods Enzymol..

[40] Winn MD (2011). Overview of the CCP4 suite and current developments. Acta Crystallogr D. Biol. Crystallogr.

[41] Liebschner D (2019). Macromolecular structure determination using X-rays, neutrons and electrons: recent developments in Phenix. Acta Crystallogr D. Struct. Biol..

[42] Emsley P, Lohkamp B, Scott WG, Cowtan K (2010). Features and development of Coot. Acta Crystallogr D. Biol. Crystallogr.

[43] Williams CJ (2018). MolProbity: more and better reference data for improved all-atom structure validation. Protein Sci..

[44] Pettersen EF (2004). UCSF Chimera–a visualization system for exploratory research and analysis. J. Comput Chem..

[45] Pettersen EF (2021). UCSF ChimeraX: structure visualization for researchers, educators, and developers. Protein Sci..

[46] Takaba K, Maki-Yonekura S, Yonekura K (2020). Collecting large datasets of rotational electron diffraction with ParallEM and SerialEM. J. Struct. Biol..

[47] Zheng SQ (2017). MotionCor2: anisotropic correction of beam-induced motion for improved cryo-electron microscopy. Nat. Methods.

[48] Zivanov J, Nakane T, Scheres SHW (2020). Estimation of high-order aberrations and anisotropic magnification from cryo-EM data sets in RELION-3.1. IUCrJ.

[49] Wang, R. Y. et al. Automated structure refinement of macromolecular assemblies from cryo-EM maps using Rosetta. *Elife* **5**, 10.7554/eLife.17219 (2016). 10.7554/eLife.17219 PMC5115868 27669148

[50] Waterhouse, A. M., Procter, J. B., Martin, D. M., Clamp, M. & Barton, G. J. Jalview Version 2–a multiple sequence alignment editor and analysis workbench. *Bioinformatics* **25**, 1189–1191, (2009). 10.1093/bioinformatics/btp033 PMC2672624 19151095

