# Supplementary material for: Atomic structure of the predominant GII.4 human norovirus capsid reveals novel stability and plasticity
Source: Nat Commun. 2022 Mar 10;13:1241. doi: 10.1038/s41467-022-28757-z (PMC8913647; doi:10.1038/s41467-022-28757-z)
Supplement: Supplementary file 1 — Supplementary Information [file 41467_2022_28757_MOESM1_ESM.pdf]

Supplementary Information

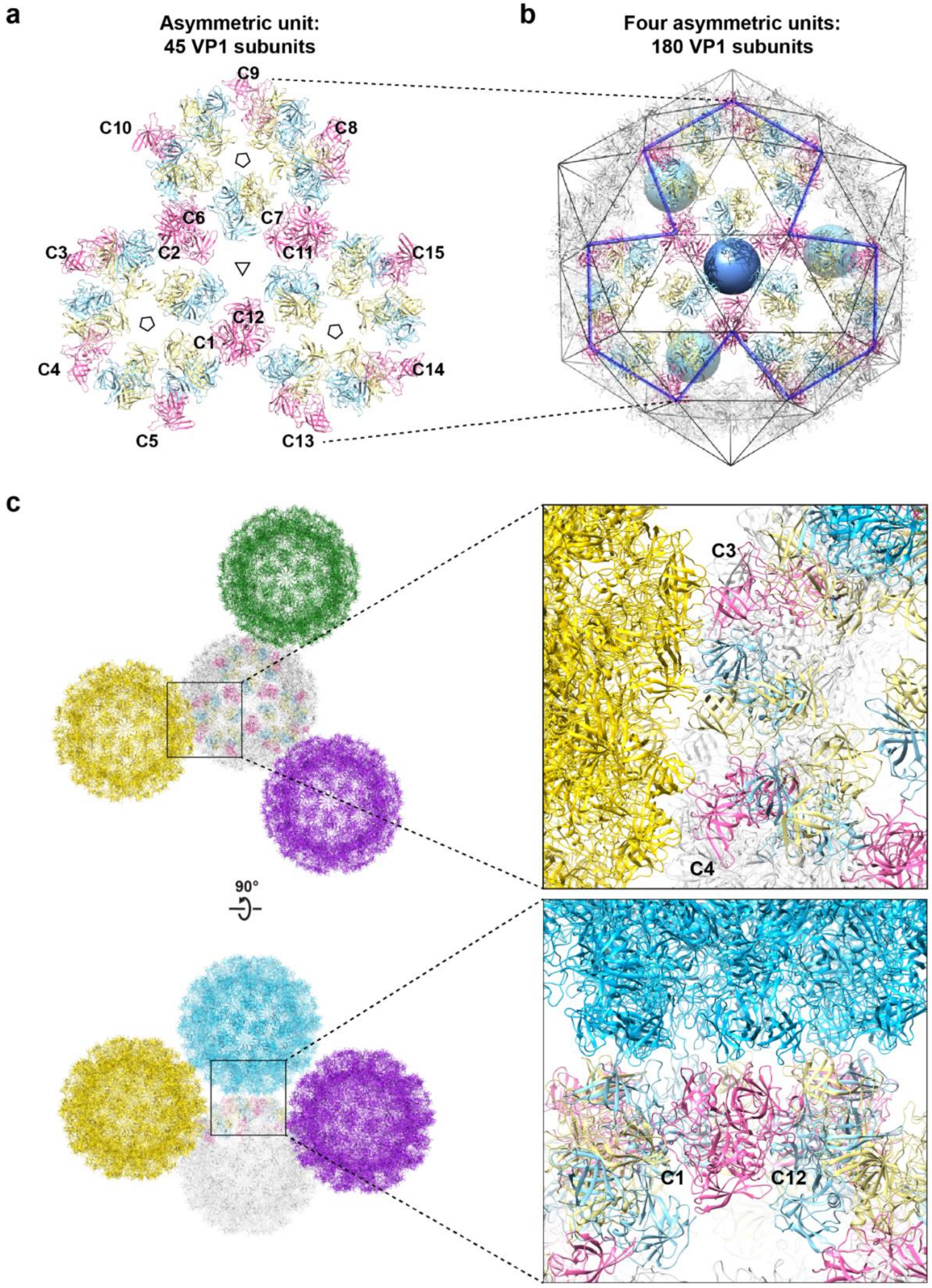

Supplementary Figure 1 | Crystal packing of GII.4 HOV VLPs. a The asymmetric unit of GII.4 HOV VLP crystal contains 15 copies of A (yellow), B (blue), and C (pink) chains. For clarity, only C subunits are labeled. The five-fold and three-fold axes are indicated by a pentagon and a triangle, respectively. **b** Four asymmetric units assemble into a VLP particle that consists of 180 copies of VP1. The icosahedron cage is shown with black lines. The symbol of each asymmetric unis is shown as a blue sphere. **c** The crystal packing of VLPs shows the C subunits interacting with the neighboring VLP particles colored in gold, green, purple, and blue.

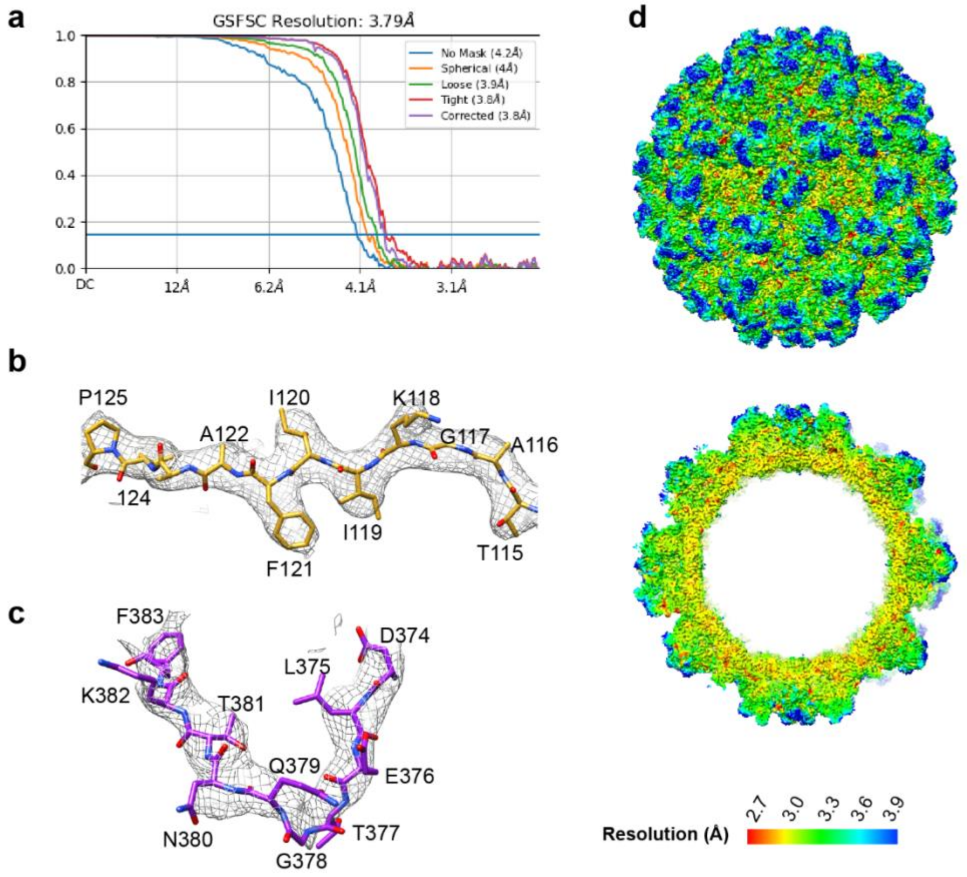

Supplementary Figure 2 | a Fourier shell correlation (FSC) curve indicates the cryo-EM structure's resolution. The resolution estimation of the reconstructions was based on the 0.143 criteria for the comparison between two half-data sets. **b-c Representative section cryo-EM map of VLP.** The density map is shown in gray mesh with the fitted atomic model of VP1. The residues are shown as stick models and labeled. **d Local resolution of the cryo-EM map.**

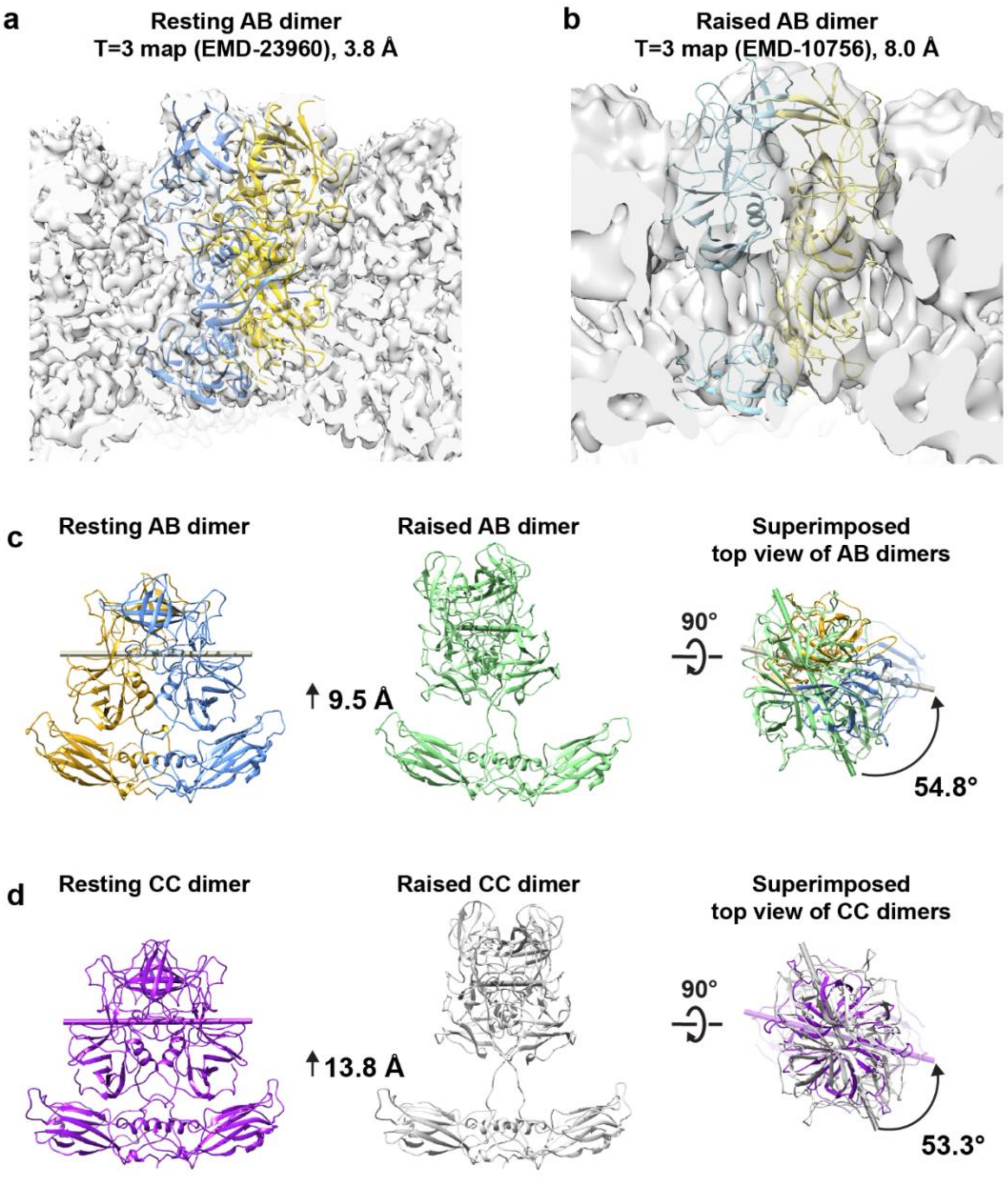

Supplementary Figure 3 | Modeling of GII.4 HOV VLP in the raised conformation. a-b GII.4 HOV VP1 fitted into the 'rising' and 'raised' conformations in the T=3 states. **c-d** Structural comparison of GII.4 HOV AB and CC dimers in both states. The P domain axis was determined by mass weighing using Chimera. The ration angles and the distance between the 'resting' and 'raised' states are calculated using Chimera.

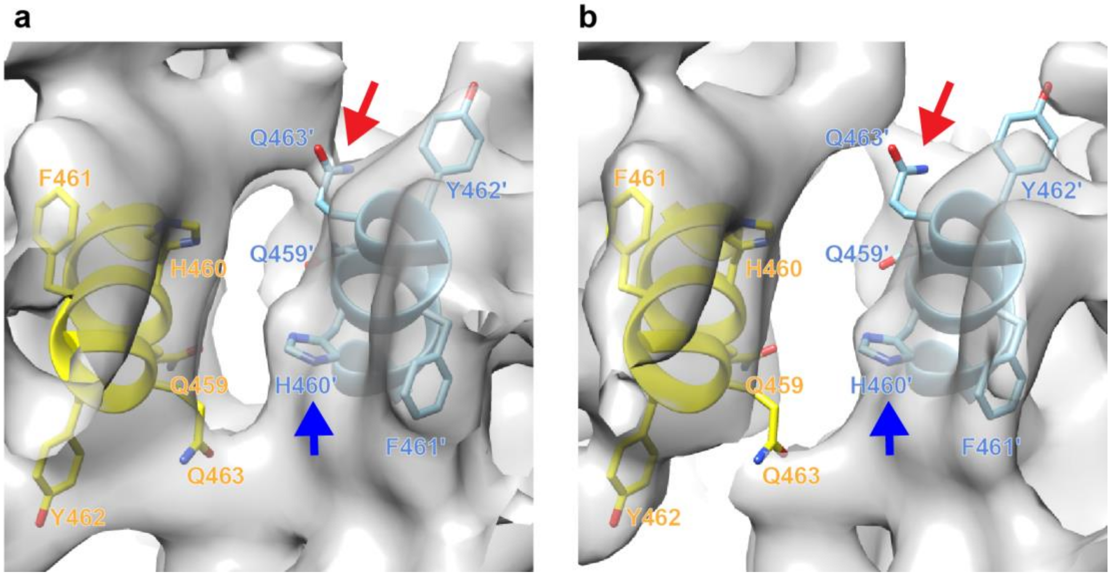

Supplementary Figure 4 | Density map of GII.4 Minerva (T = 4) VLP. Density at the VP1 dimeric interface around the residue H460 is shown in level 0.1 (**a**) and 0.14 (**b**), respectively (PDB ID: 6OUU; EMD-20206). The positions of H460 and Q463 are highlighted by blue and red arrows, respectively. No density between H460 residues was observed.

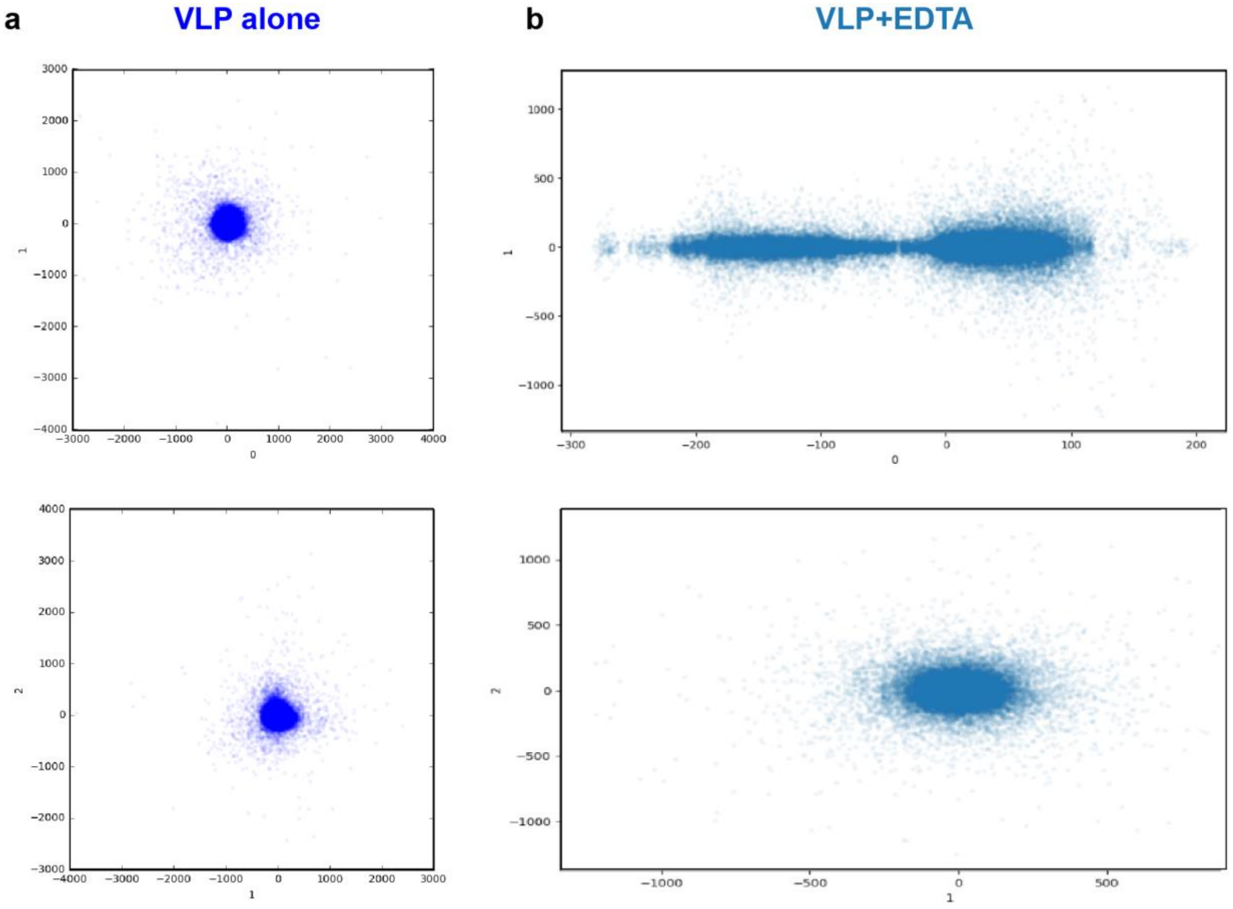

Supplementary Figure 5 | The 3D variability analysis using CryoSPARC. The reaction coordinate plots for the VLP alone (**a**) and in the presence of EDTA (**b**). The axes 0, 1, and 2, correspond to the three different components (eigenvectors) that were solved using 3DVA. The upper panel shows coordinates for 0 vs 1, and the bottom panel for 1 vs 2.

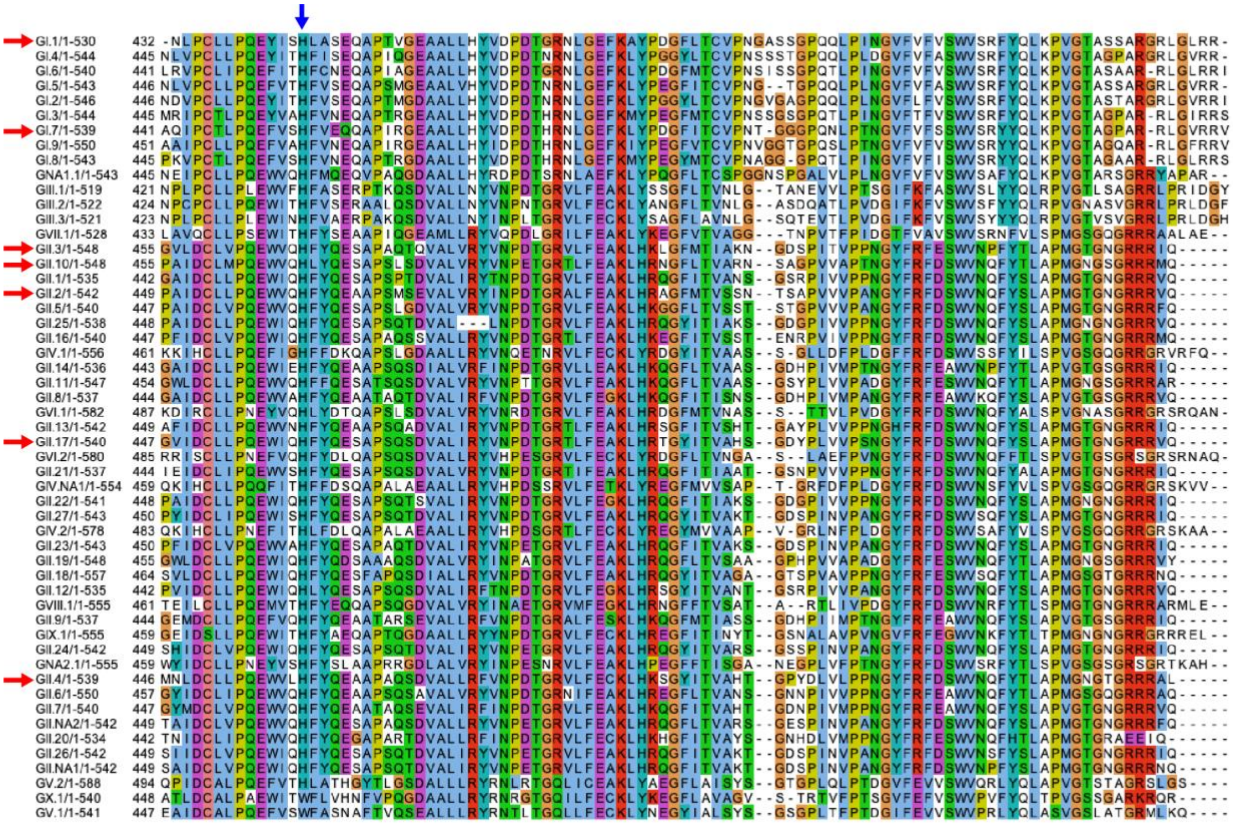

Supplementary Figure 6 | Sequence alignment of HuNoV VP1 from 49 genotypes. Multiple sequence alignment was carried using ClustalW and visualized by Jalview. The conservation of Histidine residue in all the genotypes, except GX.1 and GV.1, shown by the blue arrow at the top. The sequences we selected based on structure availability in Fig. 8 are indicated by red arrows.

Supplementary Table 1 | Human norovirus VLP structures.

Genotype	Strain	Expression	VP2	Structure	Resolution	Symmetry	P dimer	PDB or EMDB	Reference
GI.1	Norwalk	Insect cells	+	Crystal	3.4 Å	T = 3	Resting	PDB: 1IHM	Prasad (1999)
GI.1	Norwalk	Tobacco leaves	–	Cryo-EM	2.9 Å	T = 3	Resting	PDB: 6OUT<br>EMD-20199/EMD-20205	Jung (2019)
GI.7	Houston	Insect cells	+	Cryo-EM	2.9 Å	T = 3	Resting	PDB: 6OU9<br>EMD-20197/EMD-20198	Jung (2019)
GII.2	SMV	Tobacco leaves	–	Cryo-EM	3.1 Å	T = 3	Resting	PDB: 6OTF<br>EMD-20195	Jung (2019)
GII.2	SMV	Tobacco leaves	–	Cryo-EM	2.7 Å	T = 1	Resting	PDB: 6OUC<br>EMD-20201/EMD-20202	Jung (2019)
GII.4	Minerva	Tobacco leaves	–	Cryo-EM	4.1 Å	T = 4	Raised	PDB: 6OUU<br>EMD-20206	Jung (2019)
GII.4	NSW-2012	Insect cells	–	Cryo-EM	7.3 Å	T = 4	Raised	EMD-4550	Devant (2019)
GII.4	CHDC-1974	Insect cells	–	Cryo-EM	6.1 Å	T = 4	Raised	EMD-4549	Devant (2019)
GII.4	GII.4c	HEK-293T	–	Cryo-EM	4.5 Å	T = 4	Raised	EMD-10758	Devant (2021)
GII.4	GII.4c	Insect cells	–	Cryo-EM	4.2 Å	T = 4	Raised	EMD-10755	Devant (2021)
GII.4	GII.4c	Insect cells	–	Cryo-EM	8.0 Å	T = 3	Raised	EMD-10756	Devant (2021)
GII.4	GII.4c	Insect cells	–	Cryo-EM	4.2 Å	T = 1	Raised	EMD-10757	Devant (2021)
GII.10	Vietnam 026	Insect cells	–	Cryo-EM	10 Å	T = 3	Raised	EMD-5374	Hansman (2012)
GII.17	Kawasaki 2014	Insect cells	–	Cryo-EM	5.8 Å	T = 3	Raised	EMD-10759	Devant (2021)
**GII.4**	**Houston**	**Insect cells**	**+**	**Crystal**	**3.0 Å**	**T = 3**	**Resting**	**PDB: 7K6V**	**This study**
**GII.4**	**Houston**	**Insect cells**	**+**	**Cryo-EM**	**3.8 Å**	**T = 3**	**Resting**	**PDB 7MRY**<br>**EMD-23960**	**This study**

Supplementary Table 2 | X-ray crystallography data collection and refinement statistics.

PDB ID	7K6V
Wavelength (Å)	0.9795
Resolution range (Å)	29.99 - 3.0 (3.1 - 3.0)
Space group	I 2 2 2
Unit cell	
a, b, c (Å)	420.2, 446.6 464.0
α, β, γ (°)	90, 90, 90
Unique reflections	819642 (61796)
Multiplicity	
Completeness (%)	95.67 (72.59)
Mean I/sigma(I)	7.0/1.0
Wilson B-factor	67.97
R-merge (%)	13.6 (83.5)
R-work (%)	20.71 (31.13)
R-free (%)	24.18 (36.21)
Number of non-hydrogen atoms	176522
macromolecules	176443
ligands	76
solvent	3
Protein residues	22902
R.M.S. deviations	
Bond lengths (Å)	0.004
Bond angles (°)	0.77
Ramachandran	
Favored (%)	96.72
Allowed (%)	3.28
Disallowed (%)	0
Average B-factor	67.79
macromolecules	67.78
ligands	80.77
solvent	41.45

Statistics for the highest-resolution shell are shown in parentheses.

Supplementary Table 3 | Cryo-EM data collection, reconstruction, and model refinement parameters.

	HOV VLP (EMDB-23960) (PDB 7MRY)
**Data collection and processing**	
Magnification	30,000
Voltage (kV)	300
Electron exposure (e–/Å^2)	55.96
Defocus range (μm)	0.6 to 2.6
Pixel size (Å)	1.24
Symmetry imposed	I2
Map resolution (Å)	
FSC threshold	0.143
Map resolution (Å)	3.8
**Refinement**	
Initial model used (PDB code)	7K6V
Model resolution (Å)	3.8
FSC threshold	0.143
Map sharpening *B* factor (Å^2)	-210.9
Model composition	
Non-hydrogen atoms	11329
Protein residues	1460
Ligands	0
B factors (Å^2)	
Protein	72.34
Ligand	0
R.m.s. deviations	
Bond lengths (Å)	0.002
Bond angles (°)	0.529
Validation	
MolProbity score	1.45
Clashscore	5.47
Poor rotamers (%)	0
Ramachandran plot	
Favored (%)	97.11
Allowed (%)	2.89
Disallowed (%)	0
