# Supplementary material for: Atomic structure of the predominant GII.4 human norovirus capsid reveals novel stability and plasticity
Source: Nat Commun. 2022 Mar 10;13:1241. doi: 10.1038/s41467-022-28757-z (PMC8913647; doi:10.1038/s41467-022-28757-z)
Supplement: Supplementary file 3 — Description of Additional Supplementary Files [file 41467_2022_28757_MOESM3_ESM.pdf]

Description of Additional Supplementary Files

File Name: Supplementary Movie 1
Description: Shows gray density corresponding to GII.4 VLP treated with EDTA and shows the structural variability along component 0 of 3DVA.

File Name: Supplementary Movie 2
Description: Shows the same 3DVA movie overlaid with the density of untreated GII.4 VLP colored in red.
