# Supplementary material for: Atomic structure of the predominant GII.4 human norovirus capsid reveals novel stability and plasticity
Source: Nat Commun. 2022 Mar 10;13:1241. doi: 10.1038/s41467-022-28757-z (PMC8913647; doi:10.1038/s41467-022-28757-z)
Supplement: Supplementary file 6 — Reporting Summary [file 41467_2022_28757_MOESM6_ESM.pdf]

nature portfolio

Corresponding author(s): B .V. Venkataram Prasad

Last updated by author(s): 02/01/2022

Reporting Summary

Nature Portfolio wishes to improve the reproducibility of the work that we publish. This form provides structure for consistency and transparency in reporting. For further information on Nature Portfolio policies, see our Editorial Policies and the Editorial Policy Checklist.

Statistics

For all statistical analyses, confirm that the following items are present in the figure legend, table legend, main text, or Methods section.

n/a	Confirmed	
☐	☒	The exact sample size (*n*) for each experimental group/condition, given as a discrete number and unit of measurement
☐	☒	A statement on whether measurements were taken from distinct samples or whether the same sample was measured repeatedly
☒	☐	The statistical test(s) used AND whether they are one- or two-sided<br>*Only common tests should be described solely by name; describe more complex techniques in the Methods section.*
☒	☐	A description of all covariates tested
☒	☐	A description of any assumptions or corrections, such as tests of normality and adjustment for multiple comparisons
☐	☒	A full description of the statistical parameters including central tendency (e.g. means) or other basic estimates (e.g. regression coefficient) AND variation (e.g. standard deviation) or associated estimates of uncertainty (e.g. confidence intervals)
☒	☐	For null hypothesis testing, the test statistic (e.g. *F*, *t*, *r*) with confidence intervals, effect sizes, degrees of freedom and *P* value noted<br>*Give P values as exact values whenever suitable.*
☒	☐	For Bayesian analysis, information on the choice of priors and Markov chain Monte Carlo settings
☒	☐	For hierarchical and complex designs, identification of the appropriate level for tests and full reporting of outcomes
☒	☐	Estimates of effect sizes (e.g. Cohen's *d*, Pearson's *r*), indicating how they were calculated

Our web collection on statistics for biologists contains articles on many of the points above.

Software and code

Policy information about availability of computer code

Data collection	Facilities used for X-ray crystal data and cryo-EM data are noted in the text, HKL2000 was used for data collection
Data analysis	Software used for crystallography (HKL2000, CCP4, Phaser version 2.7.16, ROSETTA 3.12, PHENIX,1.19.2, and COOT version 0.9), ChimeraX Version 1.3, cryo-EM (RELION2 and cryo-SPARC3.2), 3DVA available as part of cryo-SPARC, sequence comparisons (ClustalW 1.2.2 and Jalview 2.11), DLS and BIS-Ans (Zetasizer software version 3.30, softmax Pro v5,4, and GraphPad Prism v7.0) are reported in the main text/figure legends with appropriate references.

For manuscripts utilizing custom algorithms or software that are central to the research but not yet described in published literature, software must be made available to editors and reviewers. We strongly encourage code deposition in a community repository (e.g. GitHub). See the Nature Portfolio guidelines for submitting code & software for further information.

Data

Policy information about availability of data

All manuscripts must include a data availability statement. This statement should provide the following information, where applicable:

- Accession codes, unique identifiers, or web links for publicly available datasets
- A description of any restrictions on data availability
- For clinical datasets or third party data, please ensure that the statement adheres to our policy

Atomic coordinates and structure factors for the crystal structure of the GII.4 HOV VLP have been deposited in the Protein Data Bank with the accession code 7K6V. The cryo-EM structure of GII.4 HOV VLP has been deposited in the Protein Data Bank with the accession code PDB ID 7MRY and the Electron Microscopy Databank with the accession code EMD-23960. Source data are provided with this paper.

Field-specific reporting

Please select the one below that is the best fit for your research. If you are not sure, read the appropriate sections before making your selection.

[x] Life sciences [ ] Behavioural & social sciences [ ] Ecological, evolutionary & environmental sciences

For a reference copy of the document with all sections, see nature.com/documents/nr-reporting-summary-flat.pdf

Life sciences study design

All studies must disclose on these points even when the disclosure is negative.

Sample size	we performed three technical replicates as noted in the manuscript
Data exclusions	no data were excluded
Replication	biochemical assays were replicated
Randomization	no randomization was done
Blinding	no blinding was necessary

Reporting for specific materials, systems and methods

We require information from authors about some types of materials, experimental systems and methods used in many studies. Here, indicate whether each material, system or method listed is relevant to your study. If you are not sure if a list item applies to your research, read the appropriate section before selecting a response.

Materials & experimental systems

n/a	Involved in the study	
[ ]	[x]	Antibodies
[ ]	[x]	Eukaryotic cell lines
[x]	[ ]	Palaeontology and archaeology
[x]	[ ]	Animals and other organisms
[x]	[ ]	Human research participants
[x]	[ ]	Clinical data
[x]	[ ]	Dual use research of concern

Methods

n/a	Involved in the study	
[x]	[ ]	ChIP-seq
[x]	[ ]	Flow cytometry
[x]	[ ]	MRI-based neuroimaging

Antibodies

Antibodies used	Fab portion of an antibody NORO-320 (from Dr. Jim Crowe) obtained as described in our paper Alvarado et al. Nature Communications, 2021, https://doi.org/10.1038/s41467-021-24649-w
Validation	n/a

Eukaryotic cell lines

Policy information about cell lines

Cell line source(s)	HuNoV GII.4 VLP was expressed in Gibco Sf9 insect cells using Baylor College of Medicine core facility as noted in the manuscript
Authentication	Not-authenticated
Mycoplasma contamination	cell lines were tested for mycoplasma contamination in a regular basis and found to negative
Commonly misidentified lines (See ICLAC register)	none
