# Supplementary material for: Atomic structure of the predominant GII.4 human norovirus capsid reveals novel stability and plasticity
Source: Nat Commun. 2022 Mar 10;13:1241. doi: 10.1038/s41467-022-28757-z (PMC8913647; doi:10.1038/s41467-022-28757-z)
Supplement: Supplementary file 2 — Peer Review File [file 41467_2022_28757_MOESM2_ESM.pdf]

nature portfolio

Peer Review File

Atomic structure of the predominant GII.4 human norovirus capsid reveals novel stability and plasticity

Open Access This file is licensed under a Creative Commons Attribution 4.0 International License, which permits use, sharing, adaptation, distribution and reproduction in any medium or format, as long as you give appropriate credit to the original author(s) and the source, provide a link to the Creative Commons license, and indicate if changes were made. In the cases where the authors are anonymous, such as is the case for the reports of anonymous peer reviewers, author attribution should be to 'Anonymous Referee' followed by a clear attribution to the source work. The images or other third party material in this file are included in the article's Creative Commons license, unless indicated otherwise in a credit line to the material. If material is not included in the article's Creative Commons license and your intended use is not permitted by statutory regulation or exceeds the permitted use, you will need to obtain permission directly from the copyright holder. To view a copy of this license, visit http://creativecommons.org/licenses/by/4.0/.

Reviewers' Comments:

Reviewer #1:
Remarks to the Author:
The manuscript entitled "Atomic structure of the predominant GII.4 human norovirus capsid reveals novel stability and plasticity" by Hu et al describes the details from the near atomic resolution structures determined by both X-ray crystallography and Cryo-EM of a native form (T=3) of a GII.4 norovirus capsid for the first time. This study also revealed the ion binding site that stabilizes the P domains. This report comes from the lab, which also determined the first Norovirus (Norwalk virus) structure, carefully analyzes the structure in the view of the currently available Norovirus structures that display different (raised) conformation of the P-domains.

Though well-written, the manuscript can be further improved by providing the requested information and addressing the following comments.

1) Line 111: Although the minor protein VP2 was co-expressed with VP1 to obtain VLPs…..
Even though the authors co-expressed VP1 and VP2, they don't see VP2 in their structures, which is understandable. However, it would be useful to comment on number of copies of VP2 that may be present in each particle. Also include a SDS PAGE gel showing that VP2s are indeed present in the VLPs used in the study.

2) Line 364: The supernatant containing VLPs was then pelleted by centrifugation at 12,000 rpm for 30 minutes. The pellet was then resuspended in PBS and added to a 30% sucrose cushion and pelleted again by centrifuging at 26,000 rpm for 3 hours.
Can VLPs be pelleted down in 30 mins at 12,000 rpm? There is something missing here that I don't understand.

3) Fig. 1e . I don't see the purpose of panel-e showing the capsid formed by the shell domain alone. It is confusing and also the legend is poorly written for this panel. The same goes for the Fig. 3e. I suggest removing these panels, which serve no particular purpose.

4) Fig. 3d. What are the two different "N" s indicated in the figure?

5) Fig. 7. Do these figures actually represent superpositions as the caption says?

6) Obviously, one cannot directly compare the resting and raised CP-dimers. Authors are encouraged to provide the details (e.g., RMSD) reflecting the structural similarity between the corresponding shell domain dimers and P-domain dimers in the resting and raised conformations by separating them at the hinge region.

7) Fig. 8. The a.a. sequence alignment indicates the equivalents of His460 that coordinates the divalent cation is conserved among all the Noroviruses compared. Do authors anticipate similar ion-binding in all the noroviruses? If so, why they were not seen before?

Minor:
Line 169: replace "overserved" with "observed"

Reviewer #2:
Remarks to the Author:
Hu and colleagues present the atomic structure of the GII.4 capsid in the native (T=3) state as determined by X-ray crystallography and cryo-EM. Moreover, they provide an explanation for the different conformations adopted by the capsid protein form various noroviruses reported here and

elsewhere (Devant and Hansman 2021 Virology; Song et al 2020 PLoS Pathog; Devant et al. 2019 Antivir Res; Jung et al. 2019 PNAS). Then, they compared previous structural data (Alvarado et al. 2021 Nat Comm) and the new data collected here to show that the resting P domain conformation occludes conserved epitopes and limits antibody access for binding and neutralization. This is one of the most proficient teams in the field of structural biology for gastrointestinal viruses. The data presented may be useful for the advance of our understanding of immunity to noroviruses and VLP-based vaccine development, but the discoveries represent just an incremental advance over similar publications (Devant and Hansman 2021 Virology; Alvarado et al. 2021 Nat Comm; Song et al 2020 PLoS Pathog; Devant et al. 2019 Antivir Res; Jung et al. 2019 PNAS; Lindesmith et al. 2018 mSphere).

1. The hypothesis that conformational changes affect antibody binding to the occluding epitopes is very interesting. Authors should experimentally test whether antibodies targeting those epitopes would have differential binding depending on the conformation. Immunoassays performed in buffer conditions that modify the conformations and mutagenesis of positions involved in the metal cation binding should provide such data.

2. Authors provided only a subset of genotypes for their alignment (Figure 8) to demonstrate that different residues are critical for the structural interactions among VP1 monomers. Please explain why the other genotypes have been excluded from the analyses. Are the residues discussed different among GI and GII noroviruses?

Minor comments:

1. Please provide original references for the global estimates of illness and death associated with noroviruses.

2. Neither reference #5 nor #6 seem to describe vaccine candidates under clinical development. Please use proper references for this statement.

3. Please explain the meaning of "with various epochally evolving HuNoV strains that are genetically and antigenically distinct." Are the authors suggesting that there are multiple genotypes undergoing epochal evolution? Please provide a reference for such statement.

4. Please use proper references for the statement from lines 71-72.

5. Please provide an explanation for how the authors deducted that the metal interacting with both monomers are likely Mn2+ or Mg2+ (Line 216).

Reviewer #3:
Remarks to the Author:
Hu et al. report the results of their structural analysis of the norovirus GII.4 capsid structure. X-ray and cryo-EM structures of virus-like particles (produced by recombinant expression of VP1 and VP2) are presented revealing intriguing differences that may be a consequence of crystal-packing. The structures are discussed in the context of previously published findings linking mobility of the capsid spikes (P-domains) to immune-evasion and entry pathways. The spikes of calicivirus capsids have previously been shown to rotate and lift away from the capsid surface giving rise to 'resting' and 'raised' conformations.

The structures presented here both show the P-domains in the 'resting' form, where the spikes sit close to the capsid shell and make extensive contacts. Prior publications showing the structure of GII.4 capsids were determined at lower resolution and showed a T=4 structure that is likely an

artefact as well as the likely authentic T=3 structure. In the highest published resolution structure the VLP was in a 'raised' conformation.

There is a growing body of literature that deals with conformational flexibility of norovirus spike domains (e.g. Snowden, J. S. et al. doi:10.1371/journal.pbio.3000649), of note recently the feature was linked to binding of bile-salts and hypothesised to shield neutralising epitopes in P1 and S domains (Williams et al. https://doi.org/10.1128/JVI.00176-21). Conformational flexibility is also important for receptor engagement and entry. Here only 'resting' or collapsed structures are shown. Flexibility in the spikes of the CC dimer (but not seen in the AB dimer) was found only in the X-ray structure when icosahedral symmetry was not applied, and was said to be a consequence of crystal packing constraints causing rotation of the P-domain about the hinge region. The flexibility observed indicated movement of the C-C dimer spikes to accommodate the crystal packing rather than the rotation and elevation of the spike associated with the resting to raised transition. The folds of both P and S domains remained unaltered.

In this study a metal-ion is identified at the P1 -P1 interface and postulated to induce the 'resting' state. Treatment of vlrus-like particles with chelating agent led to expansion of the capsids (measured by dynamic light scattering) and exposure of hydrophobic surfaces.

The structure determination appear to be expertly performed and I have no substantive technical criticisms. As GII.4 is an important genotype from a public health perspective, calculation of a high-resolution T=3 capsid structure is a useful accomplishment. I think that the paper would benefit from additional discussion of some (in my view) important considerations.

A key finding of the paper is that chelation of the metal ion leads to a change in hydrodynamic radius of the VLP and exposure of hydrophobic surfaces. This is proposed to indicate that the P-domains are transitioning to the raised position. Exposure of hydrophobic surfaces and increase in particle radii might also indicate a partial unfolding of the P-domain, or other loss of particle integrity. The experiment, results and interpretation appear consistent with the work of Song et al. (https://doi.org/10.1371/journal.ppat.1008619), which demonstrated that the transition from resting state to raised state could be induced by raising the pH to 8 and addition of EDTA. In that study both murine Norovirus and VLPS of GII.3 Norovirus were investigated and cryo-EM reconstruction confirmed the conformational change, albeit at lower resolution. The data presented in the present study are consistent with the literature (although pH seems to be unimportant), however the hypothesised role of the metal ion would be more convincing if a structure of the EDTA treated VLP was shown, even at modest resolution. In the discussion of the role of the metal ion - it is stated that in the published 4.1 angstrom structure of the T=4 GII.4 (raised state) VLP the ion is not present. I wonder whether it is not annotated rather than not present - model building at that resolution is more challenging, but one might expect a metal ion to be seen even at high isosurface threshold. Is it worth showing the density of the published structure in this region as well as the model? How reliable is the placement of Q463? Taken together, these data appear to support the suggestion that the metal ion is involved in stabilising the resting state, however the previous and current work are not strictly comparable. Most obviously the published structure is T=4. Further, it is not clear whether VP2 was expressed in that study, which also involved a different expression system (plant versus insect cell). Finally (at least as far as I can tell) in that study no measures were taken to chelate metal ions from the GII.4 structure, so it is unclear why it should not be present.

In line 145 it is stated that the AB dimers must strictly follow icosahedral symmetry, while the CC dimers are able to accommodate deviation from ideal symmetry. This is a confusing statement and in my view not supportable. Firstly, it is the P domains that deviate from icosahedral symmetry, not the whole VP1 (so, in the S-domain icosahedral symmetry is conserved). Secondly the CC deviations are a consequence of crystal packing. The AB P-domains may also exhibit plasticity under different packing conditions. It is certainly the case in MNV that all of the spikes deviate from icosahedral symmetry, apparently floating freely, when in a raised state (see Snowden et al).

The cryo-EM structure is presented at 3.8 angstroms resolution and is interpreted as showing that in solution the C-C dimer plasticity seen in the X-ray structure is not present. This suggestion is supported by a statement that the density of P and S domains are consistent with the achieved resolution, however density is only shown for the S-domain (residues 115-125). Was a local resolution assessment made? It would be good to show density from the P2-domain to convince readers that the CC-dimer is indeed not mobile.

The observation that the NTA was resolved in the higher-resolution X-ray structure but not in the lower-resolution cryo-EM map is intriguing. Was a protein-gel prepared for the VLPs used in the cryo-EM study? I also wonder whether this is a case of over-sharpening rather than failure to resolve density, perhaps blurring of the map might reveal this feature? Also, was the NTA density present in the X-ray data when applying full icosahedral symmetry (or is this loss of NTA information a consequence of another deviation from icosahedral symmetry?)?

Line 192 and 295 - is there any evidence that the P-S interface stabilises the capsid? Why assert that the raised form is less stable? Are there any mutagenesis/biophysics experiments to support the assertion that the noted interactions lead to a more stable capsid? Perhaps integrating a heat component to the bis-ANS +/- EDTA experiment might provide evidence for this? Considering the extensive S/NTA interactions between protomers, the role of P-S interactions would seem likely to be modest.

Line 325 - The role of structural transitions in entry pathways is mentioned for rotavirus, influenza virus and coronavirus - to support the assertion that spike mobility is functionally relevant in Norovirus, but conspicuous by its absence in this paper is discussion of the role of P-domain rotation in feline calicivirus - where the VP2 channel assembles onto the P-domains after they rotate towards the I3 axis. This is surely the most relevant study to mention (although Conley et al (2019) is cited as an example of a high-resolution animal calicivirus structure). Also VP2 is only described as being capsid associated (line 30) - why not mention the hypothesised function of this protein?

Line 148 - do you mean 'adopts'
Line 169 - do you mean 'observed'
Line 608 - do you mean 'copies'
Line 611 - I cannot see a black arrow in panel b - only in panel a (C11 vs C12)

Nature Communications manuscript NCOMMS-21-27191

We thank all the reviewers for their positive comments and suggestion. We have addressed each of the reviewers' comments in detail and revised the text accordingly. Our responses are in blue.

Responses to Reviewer #1 comments:

The manuscript entitled "Atomic structure of the predominant GII.4 human norovirus capsid reveals novel stability and plasticity" by Hu et al describes the details from the near-atomic resolution structures determined by both X-ray crystallography and Cryo-EM of a native form (T=3) of a GII.4 norovirus capsid for the first time. This study also revealed the ion-binding site that stabilizes the P domains. This report comes from the lab, which also determined the first Norovirus (Norwalk virus) structure, carefully analyzes the structure in the view of the currently available Norovirus structures that display different (raised) conformation of the P-domains.

Response: We thank the reviewer for the positive comments.

Though well-written, the manuscript can be further improved by providing the requested information and addressing the following comments.

1) Line 111: Although the minor protein VP2 was co-expressed with VP1 to obtain VLPs….. Even though the authors co-expressed VP1 and VP2, they don't see VP2 in their structures, which is understandable. However, it would be useful to comment on number of copies of VP2 that may be present in each particle. Also include a SDS PAGE gel showing that VP2s are indeed present in the VLPs used in the study.

Response: We thank the reviewer for understanding that it is challenging to visualize VP2 in the structures, particularly considering the low copy number of VP2.

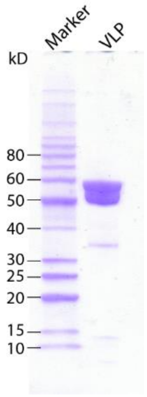

Previous studies estimated that the copy number of VP2 for Norwalk or FCV was ~1.5 - 8 using radiolabeled proteins (refs 26, 27). We previously detected VP2 in our VP1+ VP2 GI.1 VLPs using a peptide antiserum we had made (ref. 5). Unfortunately, this peptide antiserum does not cross-react with the VP2 in the GII.4 VP1+ VP2 VLPs, so we have been unable to detect VP2 directly. The baculovirus construct is the same as used for the VP1+VP2 VLPs and was confirmed by sequencing so there is no reason to think VP2 is not present in the VLPs. We are now planning to express, purify and make a new antiserum to detect GII.4 VLPs but this will not be ready to evaluate for at least 3-6 months. As this is not the major point of this paper, we hope acceptance and publication will not be delayed for this reason. SDS-PAGE gel analysis per se was not sensitive to detect VP2 (shown here for the reviewers), very likely due to the low occupancy of VP2. We have added a comment about the copy number of VP2 in VLPs and the references (page 6, lines 114-115).

2) Line 364: The supernatant containing VLPs was then pelleted by centrifugation at 12,000 rpm for 30 minutes. The pellet was then resuspended in PBS and added to a 30% sucrose cushion and pelleted again by centrifuging at 26,000 rpm for 3 hours.

Can VLPs be pelleted down in 30 mins at 12,000 rpm? There is something missing here that I don’t understand.

Response: We thank the reviewer for noticing this error. We have corrected the method section (page 18, lines 392-396): "The supernatant containing VLPs was then clarified by centrifugation at 12,000 rpm for 30 minutes to remove aggregates and cell debris. Next, the clarified supernatant was transferred to a fresh tube, and a 30% sucrose cushion was gently added to the bottom of the tube. This sample containing the 30% cushion was then centrifuged at 26,000 rpm for 3 hours to pellet the VLPs."

3) Fig. 1e . I don’t see the purpose of panel-e showing the capsid formed by the shell domain alone. It is confusing and also the legend is poorly written for this panel. The same goes for the Fig. 3e. I suggest removing these panels, which serve no particular purpose.

Response: We thank the reviewer for the suggestion. We have removed the panels showing the shell domain alone and the corresponding figure legends (page 28, fig.1 and page 30, fig.3).

4) Fig. 3d. What are the two different “N” s indicated in the figure?

Response: The black and colored "N"s indicate the N-terminus of VP1 in the crystal and cryo-EM structures, respectively. We have edited the figure legend to clarify this (page 30, lines 697-699).

5) Fig. 7. Do these figures actually represent superpositions as the caption says?

Response: Yes. We confirm that these figures show the superimposed structures side-by-side with the same scale and orientations.

6) Obviously, one cannot directly compare the resting and raised CP-dimers. Authors are encouraged to provide the details (e.g., RMSD) reflecting the structural similarity between the corresponding shell domain dimers and P-domain dimers in the resting and raised conformations by separating them at the hinge region.

Response: We thank the reviewer for the suggestion. We have compared the S and P domains separately and calculated the RMSDs. And we have added the RMSD values in the revised manuscript (page 14, lines 308-309).

7) Fig. 8. The a.a. sequence alignment indicates the equivalents of His460 that coordinates the divalent cation is conserved among all the Noroviruses compared. Do authors anticipate similar ion-binding in all the noroviruses? If so, why they were not seen before?

Response: We don't anticipate that all noroviruses can bind to divalent ions. Subtle amino acid changes around the conserved Histidine residue could affect the microenvironment for ion-binding. For example, the L459Q mutation in GII.4 Minerva strain could affect ion-binding activity.

Minor:
Line 169: replace “overserved” with “observed”

Response: We thank the reviewer for catching the typo. We have corrected this mistake in the revised manuscript (page 9, line 174).

Responses to Reviewer #2 comments:

Hu and colleagues present the atomic structure of the GII.4 capsid in the native (T=3) state as determined by X-ray crystallography and cryo-EM. Moreover, they provide an explanation for the different conformations adopted by the capsid protein form various noroviruses reported here and elsewhere (Devant and Hansman 2021 Virology; Song et al 2020 PLoS Pathog; Devant et al. 2019 Antivir Res; Jung et al. 2019 PNAS). Then, they compared previous structural data (Alvarado et al. 2021 Nat Comm) and the new data collected here to show that the resting P domain conformation occludes conserved epitopes and limits antibody access for binding and neutralization. This is one of the most proficient teams in the field of structural biology for gastrointestinal viruses. The data presented may be useful for the advance of our understanding of immunity to noroviruses and VLP-based vaccine development, but the discoveries represent just an incremental advance over similar publications (Devant and Hansman 2021 Virology; Alvarado et al. 2021 Nat Comm; Song et al 2020 PLoS Pathog; Devant et al. 2019 Antivir Res; Jung et al. 2019 PNAS; Lindesmith et al. 2018 mSphere).

1. The hypothesis that conformational changes affect antibody binding to the occluding epitopes is very interesting. Authors should experimentally test whether antibodies targeting those epitopes would have differential binding depending on the conformation. Immunoassays performed in buffer conditions that modify the conformations and mutagenesis of positions involved in the metal cation binding should provide such data.

Response: We thank the reviewer for suggesting this interesting point. We have now added new data from our biolayer interferometry (BLI) studies, which shows increased binding of NORO-320 Fab to GII.4 HOV VLP in the presence of EDTA (see Fig. 6d), which supports our hypothesis. (page 11, lines 233-238 and page 34, new figure 6d)

2. Authors provided only a subset of genotypes for their alignment (Figure 8) to demonstrate that different residues are critical for the structural interactions among VP1 monomers. Please explain why the other genotypes have been excluded from the analyses.

Response: We thought including all the 49 genotypes in the sequence alignment with a readable font would be difficult and hence included only the alignment of the VP1 sequences for which structures are available to illustrate the sequence-structure relationships. We have now attached the alignment with 49 genotypes here as per Reviewer's suggestion (See new supplemental figure Fig. S6 on page 46)

Are the residues discussed different among GI and GII noroviruses?

Response: Yes. The residues discussed are different among GI and GII noroviruses. We have included the GI.1 Norwalk and GI.7 TCH-060 VP1 sequences in Figure 8. Although the GI noroviruses contain a conserved His460, subtle sequence changes are in the adjacent residues. For example, Leu460 was mutated to Serine, and Y462 was changed to Valine.

Minor comments:

1. Please provide original references for the global estimates of illness and death associated with noroviruses.

Response: We have added the original references for the global estimates (page 3, line 27)

- Kirk, M. D. et al. World Health Organization Estimates of the Global and Regional Disease Burden of 22 Foodborne Bacterial, Protozoal, and Viral Diseases, 2010: A Data Synthesis. PLoS Med 12, e1001921, doi:10.1371/journal.pmed.1001921 (2015).
- Mortality, G. B. D. & Causes of Death, C. Global, regional, and national life expectancy, all-cause mortality, and cause-specific mortality for 249 causes of death, 1980-2015: a systematic analysis for the Global Burden of Disease Study 2015. Lancet 388, 1459-1544, doi:10.1016/S0140-6736(16)31012-1 (2016)

2. Neither reference #5 nor #6 seem to describe vaccine candidates under clinical development. Please use proper references for this statement.

Response: We have corrected the reference (page 3, line 39)

- Sherwood, J. et al. Efficacy of an intramuscular bivalent norovirus GI.1/GII.4 virus-like particle vaccine candidate in healthy US adults. Vaccine 38, 6442-6449, doi:10.1016/j.vaccine.2020.07.069 (2020).
- Kim, L. et al. Safety and immunogenicity of an oral tablet norovirus vaccine, a phase I randomized, placebo-controlled trial. JCI Insight 3, doi:10.1172/jci.insight.121077 (2018).

3. Please explain the meaning of "with various epochally evolving HuNoV strains that are genetically and antigenically distinct." Are the authors suggesting that there are multiple genotypes undergoing epochal evolution? Please provide a reference for such statement.

Response: We have added the reference and edited this statement to " various epochally evolving GII.4 HuNoVs that are genetically and antigenically distinct. " (page 3, line 42)

- Siebenga, J. J. et al. Epochal evolution of GGII.4 norovirus capsid proteins from 1995 to 2006. J Virol 81, 9932-9941, doi:10.1128/JVI.00674-07 (2007).

4. Please use proper references for the statement from lines 71-72.

Response: We have referenced the previous study by Devant et al. for the statement that GII.4 virion exhibits T=3 symmetry. The study showed the negative stain EM images of GII.4 virion and discussed the T=3 symmetry.

- Devant, J. M., Hofhaus, G., Bhella, D. & Hansman, G. S. Heterologous expression of human norovirus GII.4 VP1 leads to assembly of T=4 virus-like particles. Antiviral Res 168, 175-182, doi:10.1016/j.antiviral.2019.05.010 (2019)

We have added another earlier publication where T=1 and T=3 virions from stool samples have been observed.

- Taniguchi, K., Urasawa, S. & Urasawa, T. Further studies of 35--40 nm virus-like particles associated with outbreaks of acute gastroenteritis. J Med Microbiol 14, 107-118, doi:10.1099/00222615-14-1-107 (1981).

We have edited the sentence from " a diameter of ~ 400 Å that is consistent with a T=3 symmetry " to " a smaller diameter of ~ 400 Å that is consistent with a T=3 symmetry using negative stain EM". (page 5, lines 73-74)

5. Please provide an explanation for how the authors deducted that the metal interacting with both monomers are likely Mn2+ or Mg2+ (Line 216).

Response: Noted, we cannot be sure what type of metal ion from structural studies. However, it is most likely a divalent cation such as Mn^{2+}, Mg^{2+}, or Ca^{2+}, as our data show that treatment with EDTA has a pronounced effect on the structure. We have deleted the sentence (page 11, lines 223-224).

Reviewer #3:

Hu et al. report the results of their structural analysis of the norovirus GII.4 capsid structure. X-ray and cryo-EM structures of virus-like particles (produced by recombinant expression of VP1 and VP2) are presented revealing intriguing differences that may be a consequence of crystal-packing. The structures are discussed in the context of previously published findings linking mobility of the capsid spikes (P-domains) to immune-evasion and entry pathways. The spikes of calicivirus capsids have previously been shown to rotate and lift away from the capsid surface giving rise to 'resting' and 'raised' conformations.

Response: We agree with the reviewer that several studies have previously shown murine norovirus capsid in both 'resting' and 'raised' conformation. For human noroviruses, the VLP structures have been determined either in the 'resting' state in T=1 or T=3 or the 'raised' state in T=3 or T=4. Please see Table S1.

The structures presented here both show the P-domains in the 'resting' form, where the spikes sit close to the capsid shell and make extensive contacts. Prior publications showing the structure of GII.4 capsids were determined at lower resolution and showed a T=4 structure that is likely an artefact as well as the likely authentic T=3 structure. In the highest published resolution structure the VLP was in a 'raised' conformation.
There is a growing body of literature that deals with conformational flexibility of norovirus spike domains (e.g. Snowden, J. S. et al. doi:10.1371/journal.pbio.3000649), of note recently the feature was linked to binding of bile-salts and hypothesised to shield neutralising epitopes in P1 and S domains (Williams et al. https://doi.org/10.1128/JVI.00176-21). Conformational flexibility is also important for receptor engagement and entry.

Response: We agree with the reviewer that there were many studies with murine noroviruses. We report the first high-resolution structure of the most prevalent human norovirus GII.4 in the native T=3 state.

Here only 'resting' or collapsed structures are shown. Flexibility in the spikes of the CC dimer (but not seen in the AB dimer) was found only in the X-ray structure when icosahedral symmetry was not applied, and was said to be a consequence of crystal packing constraints causing rotation of the P-domain about the hinge region. The flexibility observed indicated movement of the C-C dimer spikes to accommodate the crystal packing rather than the rotation and elevation of the spike associated with the resting to raised transition. The folds of both P and S domains remained unaltered.

In this study a metal-ion is identified at the P1 -P1 interface and postulated to induce the 'resting' state. Treatment of vlrus-like particles with chelating agent led to expansion of the capsids (measured by dynamic light scattering) and exposure of hydrophobic surfaces. The structure determination appear to be expertly performed and I have no substantive technical criticisms. As GII.4 is an important genotype from a public health perspective, calculation of a high-resolution T=3 capsid structure is a useful accomplishment.

Response: We thank the reviewer for the positive comments.

I think that the paper would benefit from additional discussion of some (in my view) important considerations.

A key finding of the paper is that chelation of the metal ion leads to a change in hydrodynamic radius of the VLP and exposure of hydrophobic surfaces. This is proposed to indicate that the P-domains are transitioning to the raised position. Exposure of hydrophobic surfaces and increase in particle radii might also indicate a partial unfolding of the P-domain, or other loss of particle integrity. The experiment, results and interpretation appear consistent with the work of Song et al. (https://doi.org/10.1371/journal.ppat.1008619), which demonstrated that the transition from resting state to raised state could be induced by raising the pH to 8 and addition of EDTA. In that study both murine Norovirus and VLPS of GII.3 Norovirus were investigated and cryo-EM reconstruction confirmed the conformational change, albeit at lower resolution. The data presented in the present study are consistent with the literature (although pH seems to be unimportant), however the hypothesised role of the metal ion would be more convincing if a structure of the EDTA treated VLP was shown, even at modest resolution.

Response: We thank the reviewer for the positive comments and suggestions. We have carried out cryo-EM studies of EDTA-treated VLP as suggested by this reviewer. (pages 11-12, lines 239-248),

The 3D variability analysis using CryoSPARC (see ref. 30 for method description). The results show that upon EDTA treatment, the particle images are split into two populations. This is clearly in contrast to 3DVA analysis of untreated VLP images, which show a single cluster. This analysis clearly shows that EDTA treatment introduces heterogeneity and variation is continuous instead of a distinct single population. This is also clearly evident in the depiction of the variability in 3-D, as seen in the movies included in supplementary data (new Fig. S5). These movies (included below for easy reference) show that raising of the P domain and the thinning of the density between the P and S domains with EDTA treatment

Supplemental_movie1.mpg Supplemental_movie2.mpg

Movie Caption: Supplemental_movie1.mpg shows gray density corresponding to GII.4 VLP treated with EDTA and shows the structural variability along component 0 of 3DVA. Supplemental_movie2.mpg shows the same 3DVA movie overlaid with the density of untreated GII.4 VLP colored in red.

In the discussion of the role of the metal ion - it is stated that in the published 4.1 Å structure of the T=4 GII.4 (raised state) VLP, the ion is not present. I wonder whether it is not annotated rather than not present - model building at that resolution is more challenging, but one might expect a metal ion to be seen even at high isosurface threshold. Is it worth showing the density of the published structure in this region as well as the model?

Response: We thank the reviewer for the suggestion. We have now shown the density of the published structure around these residues in a new figure (Fig. S4) at contour levels 0.1 and 0.14. Careful examination of the map at these levels did not show any density that may account for the ion. (page 44, lines 781-784)

How reliable is the placement of Q463?

Response: Please see Figure S4. The density of the side chain of Q463 is visible at the higher contour level of 0.14.

Taken together, these data appear to support the suggestion that the metal ion is involved in stabilising the resting state, however the previous and current work are not strictly comparable. Most obviously the published structure is T=4. Further, it is not clear whether VP2 was expressed in that study, which also involved a different expression system (plant versus insect cell).

Response: We agree with the reviewer that our 'resting' T=3 structure is not strictly comparable with the 'raised' T=4 structure. We have modeled the 'raised' T=3 structure (Figure S3) to show the possible structural changes in VP1.

Finally (at least as far as I can tell) in that study no measures were taken to chelate metal ions from the GII.4 structure, so it is unclear why it should not be present.

Response: In the previous study with GII.4 Minerva VLP, the VLPs were purchased from Kentucky Bioprocessing and purified further by size-exclusion chromatography using a Superose 6 Increase 10/300 column in 20 mM MES·OH (pH 5.75) and 50 mM NaCl. Whether the purification buffers before the SEC step contain chelating reagents, such as EDTA, was unknown.

- Ref: Jung, J. *et al.* High-resolution cryo-EM structures of outbreak strain human norovirus shells reveal size variations. *Proc Natl Acad Sci U S A* **116**, 12828-12832, doi:10.1073/pnas.1903562116 (2019).

Furthermore, a hydrophilic residue Q459 replaces L459 in GII.4 Minerva and GII.4 315 Sydney (Fig.8).

In line 145 it is stated that the AB dimers must strictly follow icosahedral symmetry, while the CC dimers are able to accommodate deviation from ideal symmetry. This is a confusing statement and in my view not supportable. Firstly, it is the P domains that deviate from icosahedral symmetry, not the whole VP1 (so, in the S-domain icosahedral symmetry is conserved). Secondly the CC deviations are a consequence of crystal packing. The AB P-domains may also exhibit plasticity under different packing conditions. It is certainly the case in

MNV that all of the spikes deviate from icosahedral symmetry, apparently floating freely, when in a raised state (see Snowden et al).

The cryo-EM structure is presented at 3.8 angstroms resolution and is interpreted as showing that in solution the C-C dimer plasticity seen in the X-ray structure is not present. This suggestion is supported by a statement that the density of P and S domains are consistent with the achieved resolution, however density is only shown for the S-domain (residues 115-125). Was a local resolution assessment made? It would be good to show density from the P2-domain to convince readers that the CC-dimer is indeed not mobile.

Response: We thank the reviewer for the suggestions. We have added new figures (Page 42):
Figure S2c: the density of the P2 domain of chain C.
Figure S2d: the local resolution map.

The observation that the NTA was resolved in the higher-resolution X-ray structure but not in the lower-resolution cryo-EM map is intriguing. Was a protein-gel prepared for the VLPs used in the cryo-EM study?

Response: We have prepared a figure of SDS-PAGE gel for VLPs, as shown above. We observed a band with a lower molecular weight. A recent study has shown that "N-terminal VP1 Truncations Favor T = 1 Norovirus-Like Particles" Consistently, we have also observed T=1 particle on the grid. The VLP for crystal and cryo-EM structure determination was prepared using the same protocol.

- Ref: Pogan et al., N-terminal VP1 Truncations Favor T = 1 Norovirus-Like Particles. Vaccines. 2020. Dec 24;9(1):8. doi: 10.3390/vaccines9010008.

I also wonder whether this is a case of over-sharpening rather than failure to resolve density, perhaps blurring of the map might reveal this feature?

Response: We thank the reviewer for the suggestion. We have blurred the map and did not observe the density. Our results are consistent with the previous cryo-EM structure at 4.1 Å, where only residues 46-530 were observed.

Also, was the NTA density present in the X-ray data when applying full icosahedral symmetry (or is this loss of NTA information a consequence of another deviation from icosahedral symmetry?)?

Response: Yes. The NTA density is present in the X-ray data when applying full icosahedral symmetry. We have observed the similar NTA density in our previous crystal structure of GI.1 Norwalk VLPs (Prasad et al. X-ray crystallographic structure of the Norwalk virus capsid. Science 493 286, 287-290, doi:10.1126/science.286.5438.287 (1999))

Line 192 and 295 - is there any evidence that the P-S interface stabilises the capsid? Why assert that the raised form is less stable? Are there any mutagenesis/biophysics experiments to support the assertion that the noted interactions lead to a more stable capsid? Perhaps integrating a heat component to the bis-ANS +/- EDTA experiment might provide evidence for this?

Response: We thank the reviewer for the suggestion. We have now performed bis-ANS +/- EDTA experiments at both 25ºC and 42ºC, which showed that the EDTA treatment alone is sufficient to induce the additional hydrophobic surface exposure for bis-ANS binding. A similar trend is observed when the temperature is raised from 25ºC to 42ºC. We are providing the results for the reviewer below:

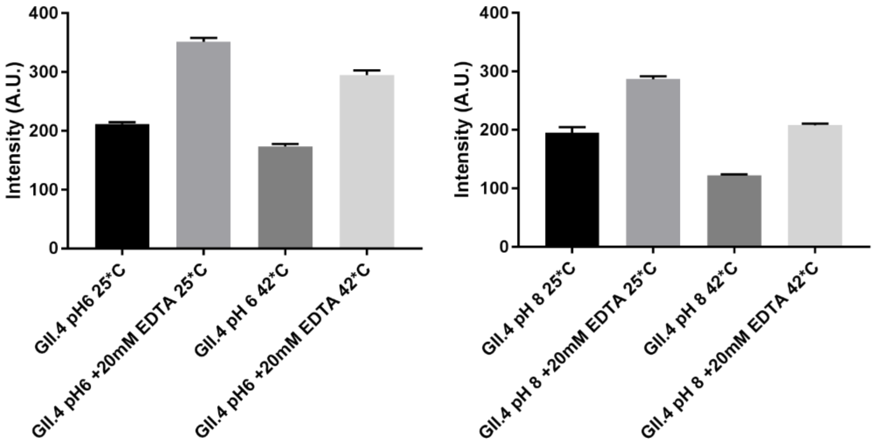

Furthermore, in the resting state, the interactions between the P and S domains are considerable, with a buried surface area of 2078 Å^2, by analyzing the interactions between S (residues 1-213) of the chain with the rest of the protein using the program PISA. The calculated G, binding energy, from the same program is -8.6 kcal/mol. In the raised state, these stabilizing interactions are lost.
We have added the discussion on page 15 (lines 318-322)

Considering the extensive S/NTA interactions between protomers, the role of P-S interactions would seem likely to be modest.

Line 325 - The role of structural transitions in entry pathways is mentioned for rotavirus, influenza virus and coronavirus - to support the assertion that spike mobility is functionally relevant in Norovirus, but conspicuous by its absence in this paper is discussion of the role of P-domain rotation in feline calicivirus - where the VP2 channel assembles onto the P-domains after they rotate towards the I3 axis. This is surely the most relevant study to mention (although Conley et al (2019) is cited as an example of a high-resolution animal calicivirus structure). Also VP2 is only described as being capsid associated (line 30) - why not mention the hypothesised function of this protein?

Response: We thank the reviewer for this remark. We have revised the text and cited Conley et al (2019) (page 16, line 351)

Although the function of VP2 in animal caliciviruses such as feline calicivirus as shown by Conley et al is suggested, any inference of the function of VP2 in HuNoVs at this stage without further studies would be very speculative.

Line 148 - do you mean 'adopts'
Line 169 - do you mean 'observed'
Line 608 - do you mean 'copies'

Response: We thank the reviewer for catching the typos. We have corrected these mistakes in the revised manuscript.
Page 8, line 152: 'adopts'
Page 9, line 174: 'observed'
Page 29, line 682: 'copies'

Line 611 - I cannot see a black arrow in panel b - only in panel a (C11 vs C12)

Response: We thank the reviewer for the remark. We have added the arrow in panel b (page 29).

Reviewers' Comments:

Reviewer #1:
Remarks to the Author:
All the criticisms, comments and suggestions of the original submission have been addressed satisfactorily.

Reviewer #2:
Remarks to the Author:
The addition of experimental data to demonstrate that VP1 conformational changes regulated by bivalent metal ions affects human antibody binding makes this paper very interesting and further research is warranted based on this new discovery.

Authors addressed all my comments and I do not have any further comment.

Reviewer #3:
Remarks to the Author:
The author responses have in the main dealt with the reviewer critique.
